# The effectiveness of sodium-glucose co-transporter 2 inhibitors on cardiorenal outcomes: an updated systematic review and meta-analysis

**DOI:** 10.1186/s12933-024-02154-w

**Published:** 2024-02-15

**Authors:** Muhammad Usman Ali, G. B. John Mancini, Donna Fitzpatrick-Lewis, Kim A. Connelly, Eileen O’Meara, Shelley Zieroth, Diana Sherifali

**Affiliations:** 1 Department of Clinical Epidemiology & Biostatistics, Faculty of Health Sciences, McMaster University, Hamilton, Canada; 2 Division of Cardiology, Centre for Cardiovascular Innovation, Department of Medicine, University of British Columbia, Vancouver, BC Canada; 3 School of Nursing, Faculty of Health Sciences, McMaster University, Hamilton, Canada; 4 Keenan Research Centre, Li Ka Shing Knowledge Institute, St. Michael’s Hospital, Toronto, ON M5B 1W8 Canada; 5 Division of Cardiology, Montreal Heart Institute, Université de Montréal, Montreal, QC Canada; 6 Section of Cardiology, Max Rady College of Medicine, University of Manitoba, Winnipeg, MB Canada

**Keywords:** Meta-analysis, Sodium-glucose co-transporter 2 inhibitors, Glucagon-like peptide-1 receptor agonists, Cardiorenal outcomes

## Abstract

**Background:**

The 2022 Canadian Cardiovascular Society (CCS) cardiorenal guideline provided clinical recommendations on sodium-glucose co-transport 2 inhibitors (SGLT2i) and glucagon-like peptide-1 receptor agonists (GLP-1RA) use. Since then, additional trials of relevance for SGLT2i have been published. This update re-evaluates the clinical recommendations for using SGLTi and their indirect comparison with existing evidence on GLP-1RA as compared to the standard of care to reduce cardiorenal morbidity and mortality.

**Methods:**

We updated our existing search and screening of the literature from September 2021 to April 2023 for randomized controlled trials of SGLT2i and GLP-1RA with placebo control. We conducted risk of bias assessment, data extraction and updated our meta-analysis of studies with similar interventions and components. The certainty of the evidence was determined using GRADE.

**Results:**

Evidence from three new trials and additional results from an updated existing trial on SGLT2i met our inclusion criteria after an updated search. Across all the included studies, the total sample size was 151,023 adults, with 90,943 in SGLT2i trials and 60,080 in GLP-1 RA trials. The mean age ranged from 59.9 to 68.4 years. Compared with standard care, the use of SGLT2i and GLP-1 RA showed significant reductions in the outcomes of cardiovascular (CV) mortality (14% & 13%), any-cause mortality (12% & 12%), major adverse CV events (MACE) (11% & 14%), heart failure (HF) hospitalization (30% & 9%), CV death or HF hospitalization (23% & 11%), and kidney composite outcome (32% & 22%). In participants with T2D, both classes demonstrated significant cardiorenal protection. But, only GLP-1RA showed a reduction in non-fatal stroke (16%) and only SGLT2i showed a reduction in HF hospitalization (30%) in this population of people living with T2D.

**Conclusions:**

This updated and comprehensive meta-analysis substantiates and strengthens the clinical recommendations of the CCS cardiorenal guidelines.

**Supplementary Information:**

The online version contains supplementary material available at 10.1186/s12933-024-02154-w.

## Introduction

The Canadian Cardiovascular Society (CCS) recently published a guideline for the use of sodium-glucose co-transporter 2 inhibitors (SGLT2i) and glucagon-like peptide-1 receptor agonists (GLP-1 RA) to reduce the risks of cardiorenal morbidity and mortality in individuals living with heart failure (HF), chronic kidney disease (CKD) and type 2 diabetes (T2D) with either concomitant atherosclerotic cardiovascular disease (ASCVD) or at high risk for ASCVD [1]. The recommendations were crafted based on a parallel, systematic review and meta-analysis published simultaneously [2]. The field has been characterized by a very rapidly expanding database of information and even since the publication of the CCS cardiorenal guideline in 2022, two additional, large, and important randomized clinical trials have been published. The first compared standard care to SGLT2i in individuals with HF and an ejection fraction (EF) > 40%, spanning both midrange EF (HFmrEF) and preserved EF (HFpEF) [3]. The second explored the value of SGLT2i in people with CKD defined by both estimated glomerular filtration rate (eGFR) and the presence or absence of elevated urinary albumin creatinine ratio (UACR) [4]. The two new studies were also relevant to our prior comparisons between SGLT2i and GLP-1 RA because they included people with and without T2D. In addition to these two large trials, results from an existing and relatively small trial “EMPULSE” that compared standard care to SGLT2i in participants hospitalized for acute heart failure have also been published [5, 6]. The decision to update the analysis of that systematic review was based on the knowledge of new publications in this field. Based on the new evidence, we sought to reconsider prior recommendations that reflect this updated and comprehensive meta-analysis.

## Methods

A full summary of our methodology was reported in an earlier publication [2 ] and this update follows the same search, screening, data extraction, and data analysis methods. We performed a comprehensive update to our existing database from September 2, 2021, to April 17, 2023. In addition, we also searched clinicaltrials.gov for any recent and relevant publications from included trials. We applied the Cochrane Risk of Bias (RoB) [7] to determine the methodological quality of those studies and extracted data for all relevant outcomes. We incorporated those data into the meta-analysis conducted previously [2] and certainty of the evidence was ascertained using Grading of Recommendations, Assessment, Development, and Evaluations (GRADE) [8].

### Data synthesis

We extracted study-reported data for primary time-to-event outcomes ((i.e., hazard ratios [HRs]) along with their 95% confidence intervals [CIs]) for CV mortality, any cause mortality, hospitalization due to HF, non-fatal MI and stroke, major cardiovascular events (MACE) and kidney composite outcomes [2] to generate the summary measures of effect using DerSimonian and Laird random-effects models with inverse variance method [9]. Serious adverse events (SEs) leading to study discontinuation is a binary outcome so we used the number of events to generate the summary measures of effect in the form of risk ratio (RR) [9]. For our quantitative synthesis, baseline population (i.e., Type 2 Diabetes, chronic kidney disease, and heart failure [HFpEF and HFrEF]) was used for primary. Further subgrouping was based on intervention type (i.e., SGLT2i and GLP-1RA). The test for sub-group differences based on intervention (SGLT2i and GLP-1RA) and heart failure population type (HFpEF and HfrEF) were also added. However, these should be interpreted cautiously as these are indirect comparisons and likely underpowered in the absence of dedicated head-to-head trials comparing the effectiveness of the two drug classes or types of heart failure populations.

We used a random-effects multi-level meta-analytic approach to account for dependency between different effect estimates based on chronic kidney disease criteria from the same study and to avoid unit of analysis error [10, 11]. We also generated summary estimates of absolute risk reduction for outcomes of interest using pooled hazard ratios and reported control group event rates. Cochran’s Q (α = 0.05) was employed to detect statistical heterogeneity and I² statistic to quantify the magnitude of statistical heterogeneity between studies where I² >50% represents moderate and I² >75% represents substantial heterogeneity across studies [12]. Funnel plots were used to assess publication bias for outcomes where there was at least 10 studies [13]. We used R software (metafor and dmetar packages) to perform all analyses [14].

## Results

**Fig. 1:**
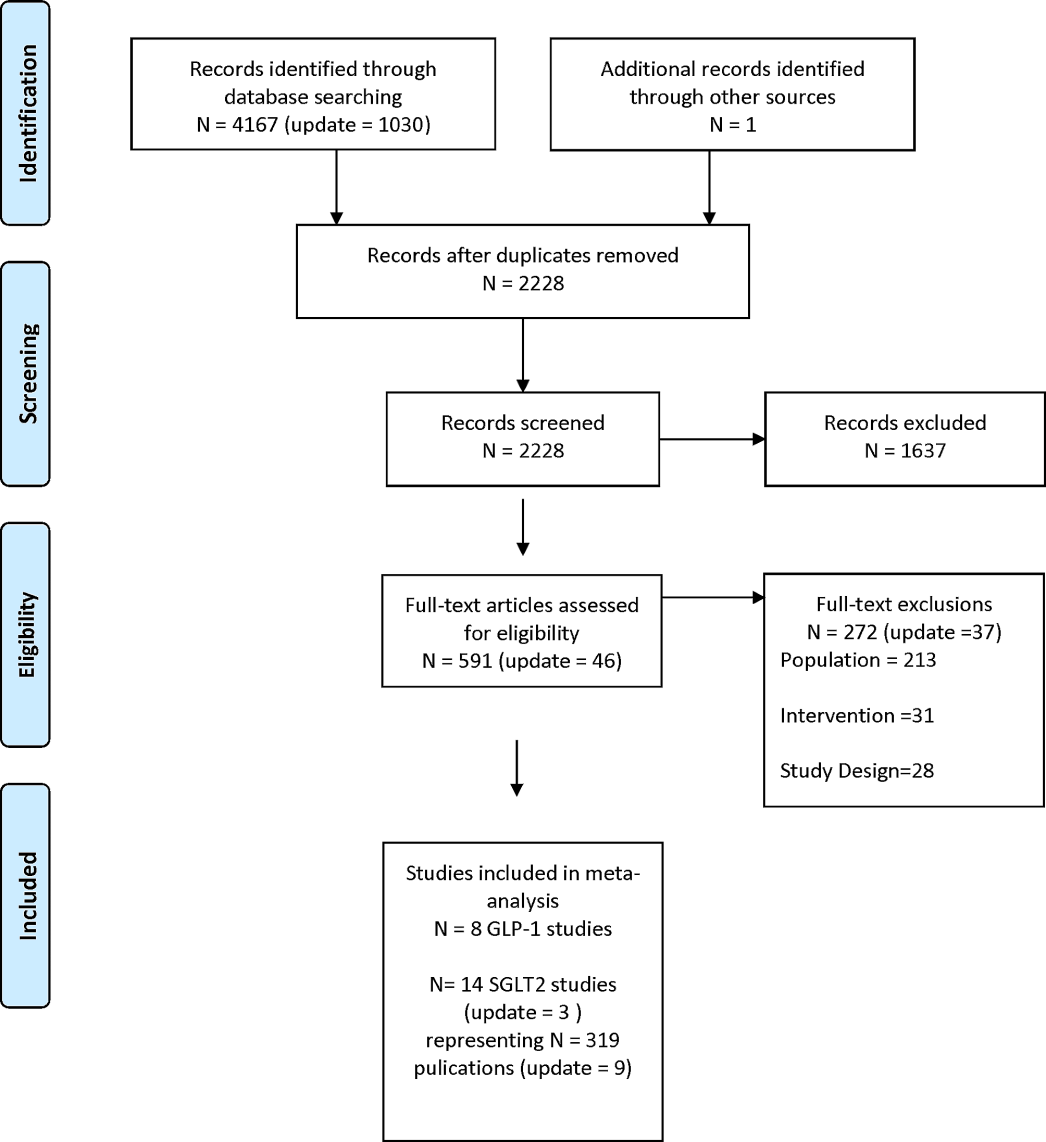
PRISMA flow diagram From: Moher D, Liberati A, Tetzlaff J, Altman DG, The PRISMA gropu (2009). Preferred reporting items for systematic reviews and meta-analyses: the PRISMA statement. PLos Med 6(6): e1000097. 10.1371/journal.pmed1000097 For more information, visit https://www.prisma-statement.org

The updated search yielded 752 additional primary papers of which 8 publications from 3 new trials on SGLT2i and 1 publication with additional results to an existing trial on SGLT2i, met the inclusion criteria (Fig. 1 Flow Diagram) [15]. Combined with the original review [2], all studies were multi-country trials; 14 studies were randomized controlled trials (RCTs) with SGLT2i treatment interventions [3, 4, 6, 16–26] and 8 studies were RCTs with GLP-1RA treatment interventions [27–34]. The total sample size across all the included studies was 151,023 adults, with 90,943 adults in SGLT2i trials and 60,080 adults in GLP-1RA trials with a mean age that ranged from 59.9 to 71.9 years. A total of 71,075 male participants were included at baseline across the trials consisting of *n* = 37,813 males for SGLT2i trials (2/14 no baseline data), and *n* = 33,262 males for GLP-1RA trials (1/8 no baseline data). Characteristics of included studies for SGLT2is are reported in Supplemental Table S1 and for GLP-1RA in our earlier publication [2].

### Risk of bias and certainty of evidence

**Fig. 2:**
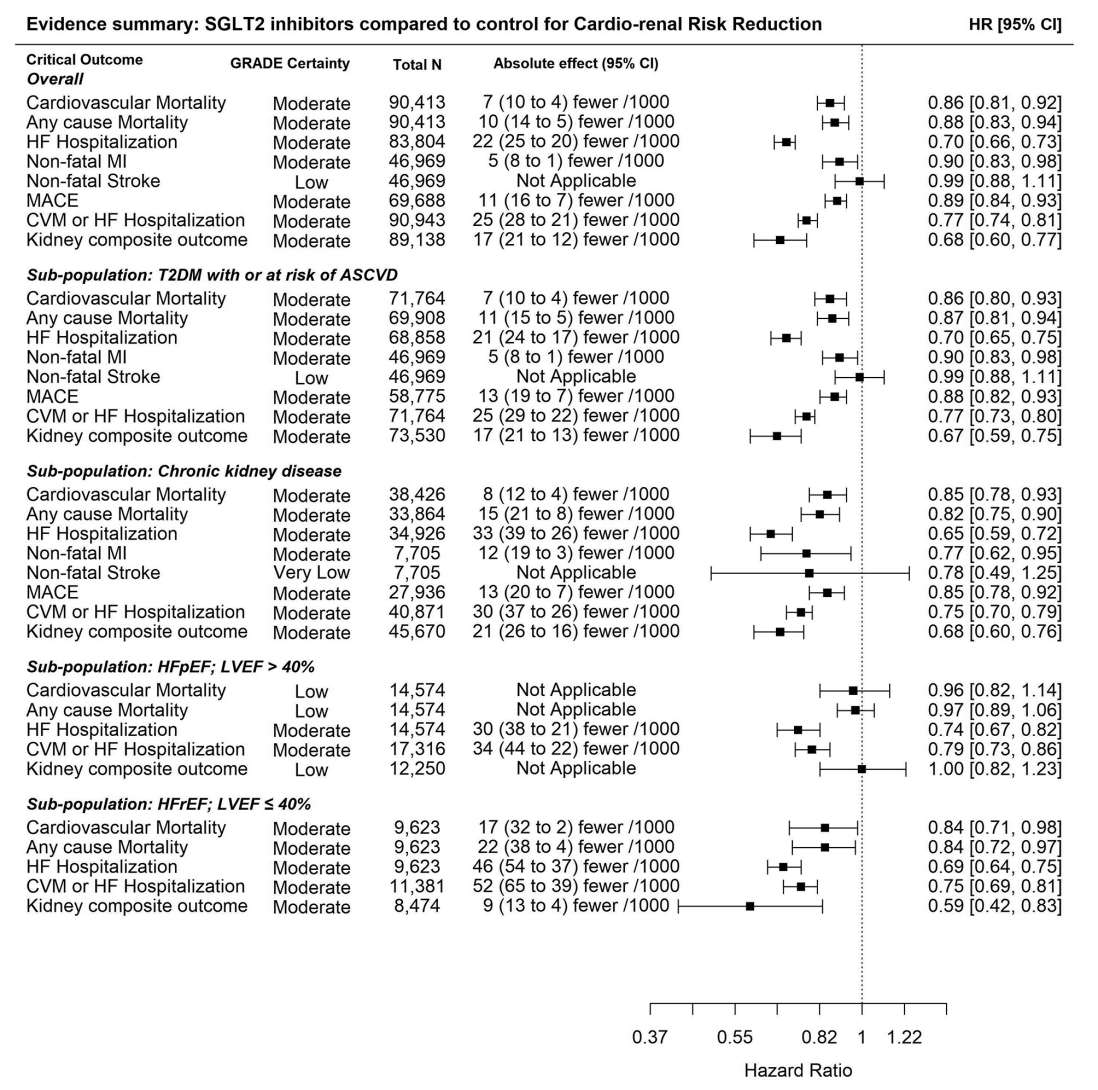
Evidence summary – SGLT2 inhibitors compared to control for cardio-renal risk reduction

The RoB for GLP-1RA has been reported in our earlier publication [2 ]. For SGLT2i, the RoB assessment resulted in 10 studies having an unclear RoB and 4 with a low RoB. Similar to our previous review [2], an unclear RoB was determined based on the lack of reporting on randomization and allocation concealment procedures; poor or no blinding of participants and/or outcome assessment; incomplete or selective outcome reporting; and other sources of bias such as industry involvement beyond funding. The addition of the studies in this update did not change GRADE for the included studies [2]. There was moderate certainty of evidence due to an unclear RoB assessment across most studies for treatment benefit outcomes, and moderate to very low certainty of the evidence depending on the subgroups (HFrEF, HFpEF, CKD and T2D) and harms outcome (Supplemental Tables S3). We present the results by outcome and subgrouped by population to facilitate an indirect comparison of effect estimates between SGLT2i and GLP-1RA in the absence of head-to-head trials. These results are summarized in Fig. 2 which provides the GRADE certainty, the absolute effect size, and the hazard ratios for updated evidence on the effectiveness of SGLT2i. Table 1 facilitates a comparison of benefits based on HR for all the cardiorenal benefits and provides a comparison between both classes of drugs when used by individuals living with T2D.

**Table 1:** Summary of hazard ratios (HR) for cardiorenal outcomes in study populations with heart failure, chronic kidney disease, or type 2 diabetes

Participant Groups		T2D	Class	MACE	All-cause mortality	CV death	Non-fatal MI	Non-fatal Stroke	HHF	CV Death or HHF	Kidney composite^¥^
**HF**	EF ≤ 40%*	**+/-**	**SGLT2i**	NA	0.84 (0.72, 0.97)	0.84 (0.71, 0.98)	NA	NA	0.69 (0.64, 0.75)	0.75 (0.69, 0.81)	0.59 (0.42, 0.83)
	**EF > 40%**	**+/-**	**SGLT2i**	NA	0.97 (0.89, 1.06)	0.96 (0.82, 1.14)	NA	NA	0.74 (0.67, 0.82)	0.79 (0.73, 0.86)	1.00 (0.82, 1.23)
**CKD**	**Any EF**	**+/-**	**SGLT2i**	0.85 (0.78, 0.92)	0.82 (0.75, 0.90)	0.85 (0.78, 0.93)	0.77 (0.62, 0.95)	0.78 (0.49, 1.25)	0.65 (0.59, 0.72)	0.75 (0.70, 0.79)	0.68 (0.60, 0.76)
		**+/-**	**GLP1-RA**	0.87 (0.75, 1.003)	0.86 (0.72, 1.02)	0.86 (0.63, 1.16)	0.86 (0.70, 1.06)	0.84 (0.51, 1.40)	0.91 (0.73, 1.15)	NA	0.85 (0.78, 0.92)
**T2D with ASCVD or multiple risk factors**	**Any EF or eGFR**	**+**	**SGLT2i**	0.88 (0.82, 0.93)	0.87 (0.81, 0.94)	0.86 (0.80, 0.93)	0.90 (0.83, 0.98)	0.99 (0.88, 1.11)	0.70 (0.65, 0.75)	0.77 (0.73, 0.80)	0.67 (0.59, 0.75)
		**+**	**GLP1-RA**	0.86 (0.80, 0.93)	0.88 (0.82, 0.94)	0.87 (0.80, 0.94)	0.94 (0.88, 1.02)	0.84 (0.76, 0.94)	0.91 (0.83, 1.002)	0.89 (0.81, 0.98)	0.78 (0.70, 0.87)

ASCVD: atherosclerotic cardiovascular disease; CKD: chronic kidney disease; CV: cardiovascular; eGFR: estimated glomerular filtration rate; GLP-1RA: glucagon-like peptide-1 receptor agonists; HHF: hospitalization for heart failure; EF: ejection fraction; MACE: major adverse cardiac events; MI: myocardial infarction; NA: not applicable; SGLT2i, sodium-glucose co-transporter 2 inhibitors; T2D, type 2 diabetes; +/-, with/without. Light green indicates a significant HR and dark green indicates a significant HR that is statistically different than in the comparator group, namely participants with EF ≤ 40% versus > 40% in SGLT2i trials of participants with HF and SGLT2i versus GLP1-RA in participants with T2D with ASCVD or multiple risk factors

*DECLARE and VERTIS CV reported results using LVEF < 45% and LVEF ≥ 45.

^**¥**^ Renal death, progression to ESKD or reduced eGFR.

### Effect on cardiorenal outcomes

The detailed results for the existing evidence on GLP-1RA have been reported in our earlier publication [2 ]. Here, we present results based on updated evidence on SGLT2is as compared to standard of care and their subsequent comparisons with effects observed for GLP-1RA.

### Participants with T2D (ASCVD/high CVD risk)

#### CV mortality

Based on moderate certainty of evidence, for the outcome of CV mortality in participants with T2D (ASCVD/high CVD risk), both SGLT2i and GLP-1RA showed a significant reduction of 14% (12 RCTS; HR = 0.86, 95% CI 0.80 to 0.93) and 13% (8 RCTS; HR = 0.87, 95% CI 0.80 to 0.94) respectively as compared to standard care. Subgroup differences for the effect on CV mortality between SGLT2i and GLP-1RA as compared with standard care in people with T2D was non-significant (*p* = 0.88) (Supplemental Figure S1).

#### Any-cause mortality

Both SGLT2i and GLP-1RA showed a significant reduction in any-cause mortality of 13% (11 RCTS; HR = 0.87, 95% CI 0.81 to 0.94) and 12% (8 RCTS; HR = 0.88, 95% CI 0.82 to 0.94) respectively as compared to standard care in participants with T2D (ASCVD/high CVD risk) based on moderate certainty of evidence. Subgroup differences for the effect on any-cause mortality between SGLT2i and GLP-1RA as compared with standard of care in participants with T2D was non-significant (*p* = 0.97) (Supplemental Figure S2).

#### CV mortality or HF hospitalization

Both SGLT2i and GLP-1RA showed a significant reduction in CV mortality or HF hospitalization of 23% (12 RCTS; HR = 0.77, 95% CI 0.73 to 0.80) and 11% (3 RCTS; HR = 0.89, 95% CI 0.81 to 0.98) respectively as compared to standard care in participants with T2D (ASCVD/high CVD risk) based on moderate certainty of evidence. Subgroup differences for the effect on CV mortality or HF hospitalization between SGLT2i and GLP-1RA as compared with standard of care in participants with T2D was significant (*p* = 0.01) with a higher benefit observed for SGLT2i (Supplemental Figure S3).

#### Hospitalization due to heart failure

In participants with T2D (ASCVD/high CVD risk), SGLT2i showed a significant reduction of 30% (11 RCTS; HR = 0.70, 95% CI 0.65 to 0.75) on hospitalization due to HF as compared to standard care based on moderate certainty of evidence. In contrast, treatment with a GLP-1RA showed no difference in effect (7 RCTS; HR = 0.91, 95% CI 0.83 to 1.002) as compared to standard care. Subgroup differences for the effect on hospitalization due to HF between SGLT2i and GLP-1RA as compared with standard care in participants with T2D was significant (*p* = 0.00001) with higher benefit observed for SGLT2i (Supplemental Figure S4).

#### Non-fatal myocardial infarction

In participants with T2D (ASCVD/high CVD risk), SGLT2i showed a significant reduction in non-fatal MI of 10% (5 RCTS; HR = 0.90, 95% CI 0.83 to 0.98) as compared to standard care; however, treatment with a GLP-1RA showed no difference in effect (7 RCTS; HR = 0.94, 95% CI 0.88 to 1.02) as compared to standard care based on moderate certainty of evidence. Subgroup differences for the effect on non-fatal MI between SGLT2i and GLP-1RA as compared with standard care in participants with T2D was non-significant (*p* = 0.42) (Supplemental Figure S5).

#### Non-fatal stroke

SGLT2i showed no difference for non-fatal stroke (5 RCTS; HR = 0.99, 95% CI 0.88 to 1.11) as compared to standard care in participants with T2D (ASCVD/high CVD risk) based on low certainty of evidence. In contrast, treatment with GLP-1RA showed a significant reduction in non-fatal stroke of 16% (7 RCTS; HR = 0.84, 95% CI 0.76 to 0.94) as compared to standard care based on moderate certainty of evidence. Subgroup differences for the effect on non-fatal stroke between SGLT2i and GLP-1RA as compared with standard care in participants with T2D was significant (*p* = 0.04) with higher benefit observed for GLP-1RA (Supplemental Figure S6).

#### Major adverse cardiac events (CV mortality, non-fatal MI, non-fatal stroke)

both SGLT2i and GLP-1RA showed a significant reduction for MACE of 12% (7 RCTS; HR = 0.88, 95% CI 0.82 to 0.93) and 14% (8 RCTS; HR = 0.86, 95% CI 0.80 to 0.93) respectively in participants with T2D (ASCVD/high CVD risk), as compared to standard care based on moderate certainty of evidence. Subgroup differences for the effect on MACE between SGLT2i and GLP-1RA as compared with standard of care in participants with T2D was non-significant (*p* = 0.87) (Supplemental Figure S7).

#### Kidney composite outcomes

In participants with T2D (ASCVD/high CVD risk), both SGLT2i and GLP-1RA showed a significant reduction in kidney composite outcomes of 33% (12 RCTS; HR = 0.67, 95% CI 0.59 to 0.75) and 22% (5 RCTS; HR = 0.78, 95% CI 0.70 to 0.87) respectively as compared to standard care based on moderate certainty of evidence. Subgroup differences for the effect on kidney composite outcomes between SGLT2i and GLP-1RA as compared with standard care in participants with T2D was marginally non-significant (*p* = 0.08) with a trend towards higher benefit for SGLT2i (Supplemental Figure S8).

### Participants with CKD

#### CV mortality

In participants with CKD, SGLT2i showed a significant reduction of 15% (10 RCTS; HR = 0.85, 95% CI 0.78 to 0.93) in CV mortality as compared to standard care based on moderate certainty of evidence. In contrast, treatment with GLP-1RA showed no difference in effect (2 RCTS; HR = 0.86, 95% CI 0.65 to 1.16). Subgroup differences for the effect on CV mortality was non-significant (*p* = 0.85) between SGLT2i and GLP-1RA as compared with standard of care in participants with CKD (Supplemental Figure S9).

#### Any-cause mortality

In participants with CKD, SGLT2i showed a significant reduction for any-cause mortality of 18% (8 RCTS; HR = 0.82, 95% CI 0.75 to 0.90) as compared to standard care based on moderate certainty of evidence. In contrast, treatment with a GLP-1RA showed no difference in effect (2 RCTS; HR = 0.86, 95% CI 0.72 to 1.02). Subgroup differences for the effect on any-cause mortality between SGLT2i and GLP-1RA as compared with standard care was non-significant (*p* = 0.65) in participants with CKD (Supplemental Figure S10).

#### CV mortality or HF hospitalization

In participants with CKD, SGLT2i showed a significant reduction of 25% (12 RCTS; HR = 0.75, 95% CI 0.70 to 0.79) for CV mortality or HF hospitalization compared to standard care (Supplemental Figure S11) based on moderate certainty of evidence. There was no data for the effect of GLP-1RA from the included studies for this outcome.

#### Hospitalization due to heart failure

In participants with CKD, SGLT2i showed a significant reduction of 35% (10 RCTS; HR = 0.65, 95% CI 0.59 to 0.72) for the outcome of hospitalization due to HF as compared to standard care based on moderate certainty of evidence. In contrast, treatment with GLP-1RA showed no difference in effect (2 RCTS; HR = 0.91, 95% CI 0.73 to 1.15). Subgroup differences for the effect on hospitalization due to HF between SGLT2i and GLP-1RA as compared with standard care in participants with CKD, was significant (*p* = 0.0001) with higher benefit observed for SGLT2i (Supplemental Figure S12).

#### Non-fatal myocardial infarction

SGLT2i showed a significant reduction of 23% (3 RCTS; HR = 0.77, 95% CI 0.62 to 0.95) in non-fatal MI as compared to standard care in participants with CKD based on moderate certainty of evidence. In contrast, treatment with GLP-1RA showed no difference in effect (2 RCTS; HR = 0.86, 95% CI 0.70 to 1.06). Subgroup differences for the effect on non-fatal MI between SGLT2i and GLP-1RA as compared with standard of care in participants with CKD was non-significant (*p* = 0.47) (Supplemental Figure S13).

#### Non-fatal stroke

Both SGLT2i (3 RCTS; HR = 0.78, 95% CI 0.49 to 1.25) and GLP-1RA (2 RCTS; HR = 0.84, 95% CI 0.51 to 1.40) showed no difference in effect for non-fatal stroke as compared to standard care in participants with CKD based on very low certainty of evidence. Subgroup differences for the effect on non-fatal stroke between SGLT2i and GLP-1RA as compared with standard of care in participants with CKD was non-significant (*p* = 0.83) (Supplemental Figure S14).

#### Major adverse cardiac events (CV mortality, non-fatal MI, non-fatal stroke)

SGLT2i showed a significant reduction in MACE of 15% (6 RCTS; HR = 0.88, 95% CI 0.78 to 0.92) as compared to standard care in participants with CKD based on moderate certainty of evidence. In contrast, treatment with a GLP-1RA showed no difference in effect (5 RCTS; HR = 0.87, 95% CI 0.75 to 1.003). Subgroup differences for the effect on MACE between SGLT2i and GLP-1RA as compared with standard of care in participants with CKD, remained non-significant (*p* = 0.74) (Supplemental Figure S15).

#### Kidney composite outcomes

In participants with CKD, both SGLT2i and GLP-1RA showed a significant reduction of 32% (12 RCTS; HR = 0.68, 95% CI 0.60 to 0.76) and 15% (3 RCTS; HR = 0.85, 95% CI 0.78 to 0.92) respectively for kidney composite outcomes as compared to standard care based on moderate certainty of evidence. Subgroup differences for the effect on kidney composite outcome between SGLT2i and GLP-1RA as compared with standard of care in participants with T2D was significant (*p* = 0.01) with a higher benefit observed for SGLT2i (Supplemental Figure S16).

### Participants with heart failure

#### CV mortality

SGLT2i showed no difference in effect (4 RCTS; HR = 0.96, 95% CI 0.82 to 1.14) for CV mortality as compared to standard care in participants with HFpEF based on low certainty of evidence. However, in participants with HFrEF, treatment with an SGLT2i showed a significant reduction of 16% (4 RCTS; HR = 0.84, 95% CI 0.71 to 0.98) as compared to standard care based on moderate certainty of evidence. Subgroup differences for the effect of SGLT2i on CV mortality in participants with HFpEF and HFrEF was non-significant (*p* = 0.22) (Supplemental Figure S17).

#### Any-cause mortality

In participants with HFpEF, SGLT2i showed no difference in effect (4 RCTS; HR = 0.97, 95% CI 0.89 to 1.06) as compared to standard care for any-cause mortality based on low certainty of evidence. In participants with HFrEF, treatment with an SGLT2i showed a significant reduction in any-cause mortality of 16% (4 RCTS; HR = 0.84, 95% CI 0.72 to 0.97) as compared to standard care based on moderate certainty of evidence. Subgroup differences for the effect of SGLT2i on any-cause mortality in participants with HFpEF and HFrEF was marginally significant (*p* = 0.05) (Supplemental Figure S18).

#### CV mortality or HF hospitalization

SGLT2i showed a significant reduction in CV mortality or HF hospitalization of 21% (6 RCTS; HR = 0.79, 95% CI 0.73 to 0.86) in participants with HFpEF and a reduction of 25% (6 RCTS; HR = 0.75, 95% CI 0.69 to 0.81) in participants with HFrEF as compared to standard care; both based on moderate certainty of evidence. Subgroup differences were non-significant (*p* = 0.32) for the effect of SGLT2i on CV mortality or HF hospitalization in participants with HFpEF and HFrEF (Supplemental Figure S19).

#### Hospitalization due to heart failure

SGLT2i showed a significant reduction in HF hospitalization of 26% (4 RCTS; HR = 0.74, 95% CI 0.67 to 0.82) in participants with HFpEF and a 31% reduction in HF hospitalization (4 RCTS; HR = 0.69, 95% CI 0.64 to 0.75) in participants with HFrEF as compared to standard care, based on moderate certainty of evidence. Subgroup differences for the effect of SGLT2i on HF hospitalization in participants with HFpEF and HFrEF was non-significant (*p* = 0.30) (Supplemental Figure S20).

#### Kidney composite outcomes

SGLT2i showed no difference in effect (2 RCTS; HR = 1.00, 95% CI 0.82 to 1.23) as compared to standard care for kidney composite outcomes in participants with HFpEF based on low certainty of evidence. In participants with HFrEF, treatment with an SGLT2i showed a significant reduction of 41% (2 RCTS; HR = 0.59, 95% CI 0.42 to 0.83) as compared to standard care based on moderate certainty of evidence. Subgroup differences in the effect of SGLT2i on kidney composite outcome in participants with HFpEF and HFrEF was significant (*p* = 0.01) with higher benefit observed in participants with HFrEF (Supplemental Figure S21).

### Serious adverse events leading to study discontinuation

Treatment with an SGLT2i showed no differences in risk of serious adverse events leading to study discontinuation (13 RCTS; RR 1.04, 95% CI 0.96 to 1.12) as compared to the standard care; however, it should be noted that the certainty of evidence was very low due to serious concerns regarding RoB, inconsistency, and imprecision. In contrast, GLP-1RA was associated with a 1.28 times higher risk of serious adverse events leading to study discontinuation as compared to standard of care (8 RCTs; RR = 1.28, 95% CI 1.04 to 1.57).

## Discussion

We provide an up-to-date meta-analysis of trials showing the benefits of SGLT2i for the reduction of cardiorenal morbidity and mortality in individuals living with HF or CKD and we reappraise the comparison of SGLT2i in people with T2D as well. In people with HF, the results show benefits across the spectrum of EF with reductions in the composite endpoint of CV Death or hospitalization for HF in the range of 21 to 25% and for reduction of hospitalization for HF in the range of 31 to 26%. However, despite the additional DELIVER trial data, reduction in all cause or CV mortality and reduction in the renal composite endpoint remain evident only in the HFrEF population (Fig. 2 and Supplemental Figures S9 – S16).

The EMPA-Kidney trial is unique in adding information to participants with CKD defined by an eGFR of at least 20 mL/min/1.73m2 but less than 45 mL/min/1.73m2 of body-surface area, or who had an eGFR of at least 45 mL/min/1.73m2 but less than 90 mL/min/1.73m2 with a urinary albumin-to-creatinine ratio (with albumin measured in milligrams and creatinine measured in grams) of at least 200. However, the report does not provide information regarding the primary or individual endpoints for these two disparate groups of participants. Subset analyses stratified by eGFR or by albuminuria separately are, strictly speaking, not heterogeneous but the report suggests that benefits are seen largely in participants with higher levels of proteinuria. We believe, therefore, that the existing CCS guidelines recommending use of an SGLT2i in adults with CKD (UACR > 20 mg/mmol, eGFR ≥ 25 mL/min/1.73m2) to reduce the composite of a significant decline in eGFR, progression to end-stage kidney disease or death due to kidney disease, all-cause and CV mortality, non-fatal MI, and hospitalization for HF remain largely unchanged although a slightly lower eGFR of 20 mL/min/1.73m2 would be considered reasonable. In this group, the endpoint of non-fatal stroke shows a point estimate reduction of 22% but this remains non-significant in the new analysis even with the new trial (Fig. 2 and Supplemental Figure S14). The effect on the combined outcome of CV mortality or HF hospitalization also remained unchanged (25% reduction) despite the data from two additional trials (DELIVER and EMPULSE, Fig. 2 and Supplemental Figure S11).

In individuals living with T2D with ASCVD or with multiple risk factors for ASCVD, the recommendations regarding the use of either SGLT2i or GLP-1 RA for reduction of all-cause or CV death or MACE are substantiated. Similarly, the inclusion of new trial data does not alter our prior conclusion that there is a strong signal for the reduction of non-fatal stroke associated with the GLP-1 but not the SGLT2i class. Additionally, the significant but modest reduction in non-fatal MI of 10% with SGLT2i remains not significantly different from the 6%-point estimate reduction associated with GLP-1 RA, which itself is not significant. Accordingly, the new data still support the reticence of the CCS cardiorenal guideline to make any recommendations regarding the reduction of non-fatal MI using SGLT2i. Finally, it is important to note that the prior CCS cardiorenal guideline committee did not feel that recommendations regarding either HF or CKD protection using GLP1-RA were warranted in the absence of published, dedicated trials in these populations. In the population of people with T2D, the signal for the reduction in the renal composite endpoint of 22% associated with GLP-1 RA use was inferior to the 35% reduction associated with the use of SGLT2i. In the current analysis, however, the risk reduction associated with SGLT2i is somewhat diminished (33%) to an extent that is no longer superior to the GLP-1 RA class. This slight statistical change, however, does not warrant any change in recommendations as it does not overcome the current absence of a dedicated CKD trial using GLP-1 RA while underscoring the importance of pending trials [35]. Publically available announcements suggest that the pending trial results [36] will demonstrate renal risk reduction, but data is not currently available. In addition, results from ongoing trials [37, 38] may also provide further insights into the role of GLP1 RAs for cardio-renal protection in the ASCVD population. Therefore, updating current meta-analyses will be warranted to consider the benefits and harms of GLP-1 RA in this context. In the meantime, it is certainly not unreasonable to consider this potential benefit of adding GLP-1 RA in people with T2D already on SGLT2i but not yet at the A1c goal. The majority of the evidence in our review included patients with chronic heart failure, with a limited number of trials identified on cardiorenal protection of SGLT2i and GLP-1 RA in acute heart failure. Including this limited evidence on acute heart failure in our quantitative synthesis may have introduced some heterogeneity across summary estimates based on the type of heart failure population.

## Conclusions

This analysis is the most up-to-date and comprehensive meta-analysis available to our knowledge regarding the benefits of SGLT2i and GLP-1 RA for cardiorenal protection in individuals living with HF, CKD and T2D with ASCVD or with risk factors for ASCVD. The results substantiate the recommendations of the CCS cardiorenal guidelines while highlighting that advances and new trials, particularly for those with CKD, may support further expansion of the use of these classes for cardiorenal protection.

### Electronic supplementary material

Below is the link to the electronic supplementary material.

Supplementary Material 1
