# Supplementary material for: The effectiveness of sodium-glucose co-transporter 2 inhibitors on cardiorenal outcomes: an updated systematic review and meta-analysis
Source: Cardiovasc Diabetol. 2024 Feb 15;23:72. doi: 10.1186/s12933-024-02154-w (PMC10870515; doi:10.1186/s12933-024-02154-w)
Supplement: Supplementary file 1 — Supplementary Material 1 [file 12933_2024_2154_MOESM1_ESM.docx]

**Supplemental Figures S1 – S8: Meta-analysis of outcomes for people with Type 2 diabetes**

**Supplemental Figure S1 – Cardiovascular mortality**


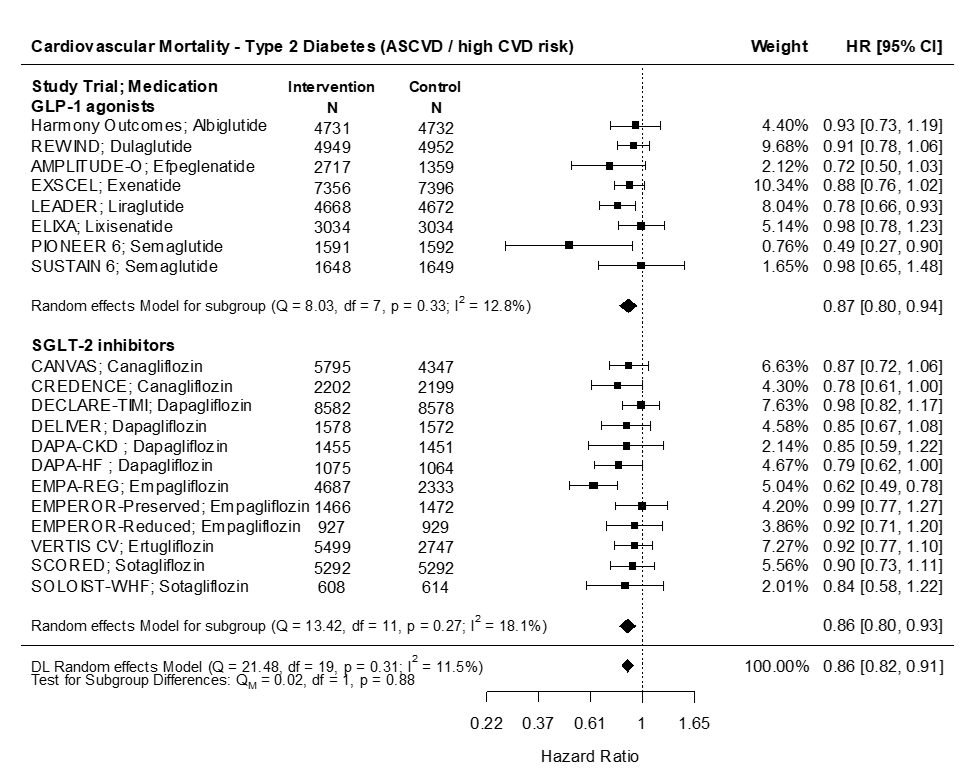


**Supplemental Figure S2 – Any-cause mortality**


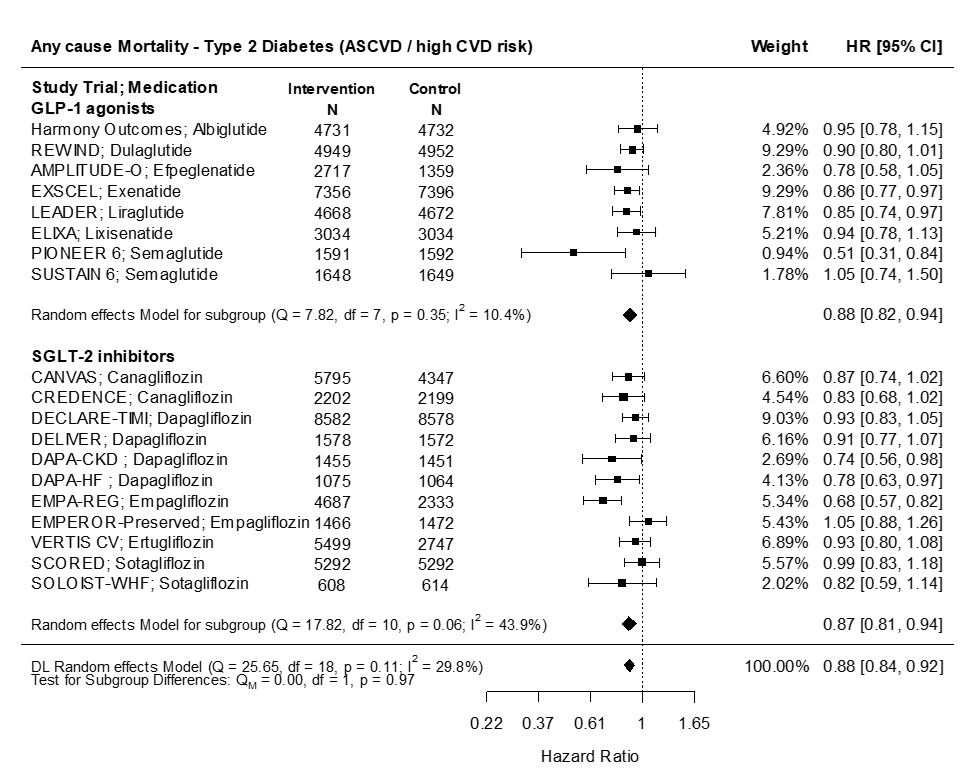


**Supplemental Figure S3 – Cardiovascular mortality or hospitalization heart failure**


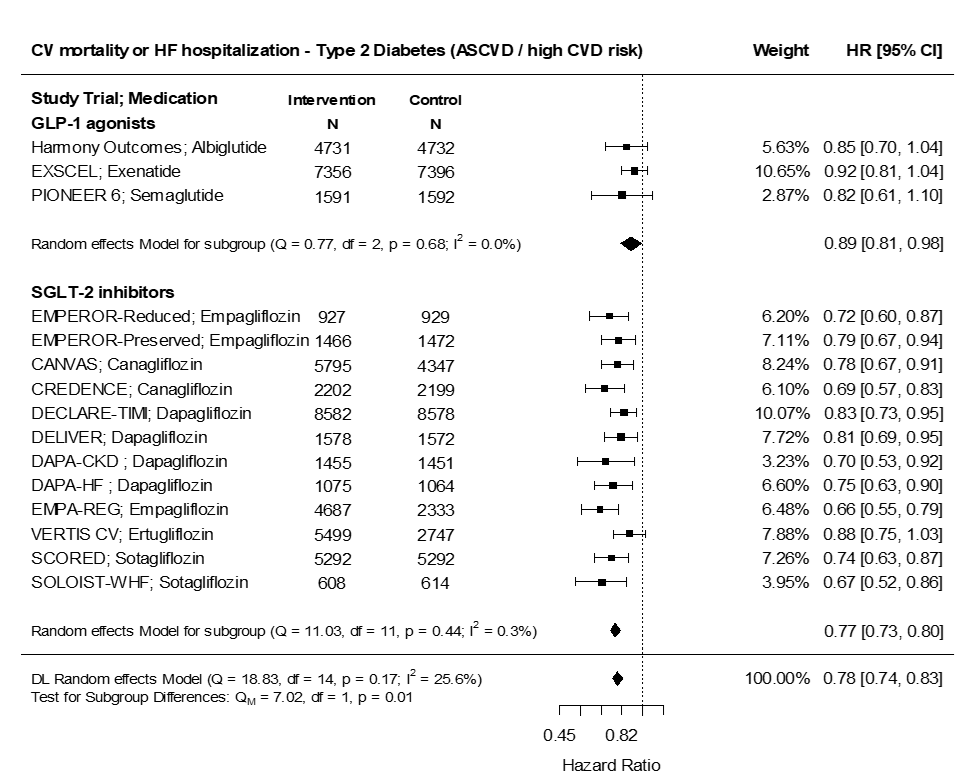


**Supplemental Figure S4 – Hospitalization due to heart failure**


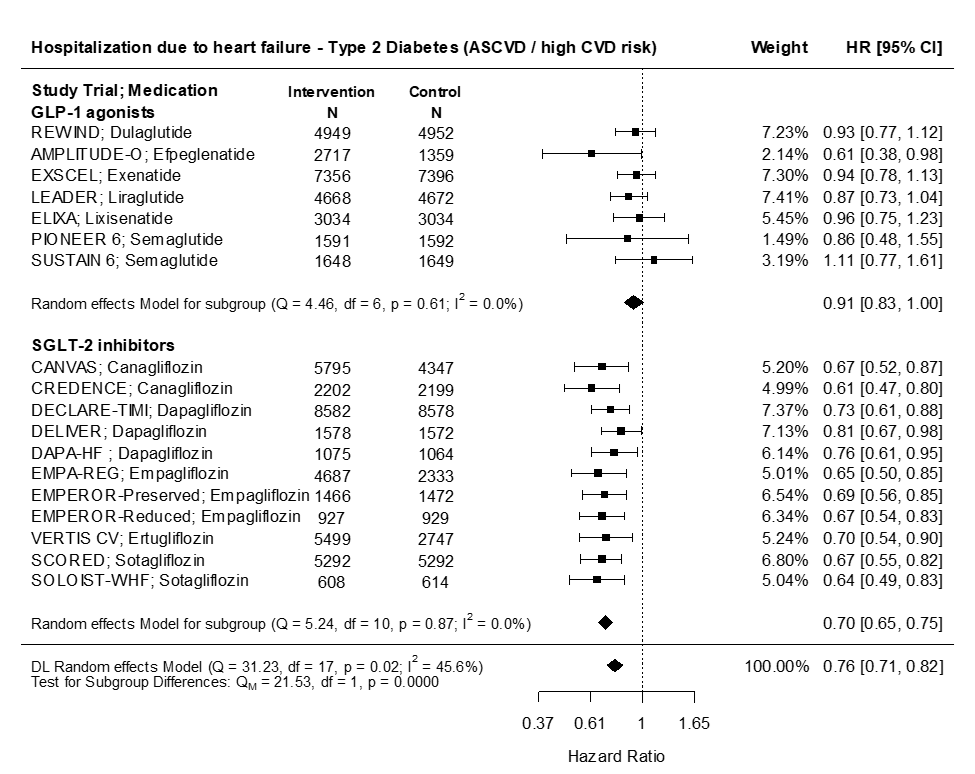


**Supplemental Figure S5 –** **Non-fatal myocardial infarction**


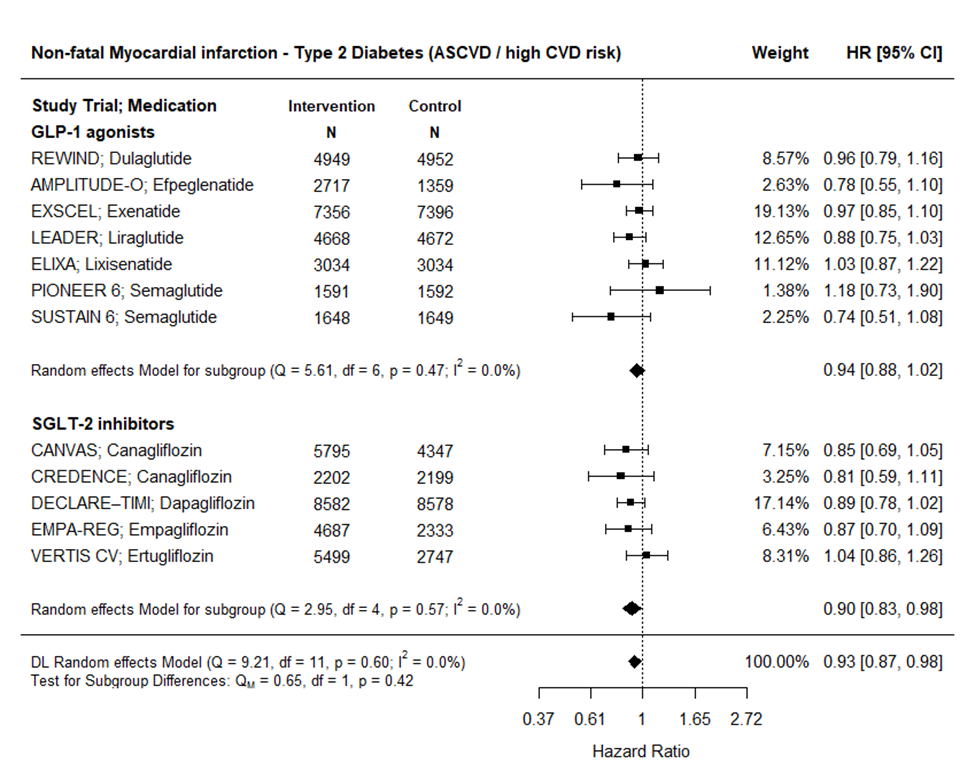


**Supplemental Figure S6 – Non-fatal stroke**


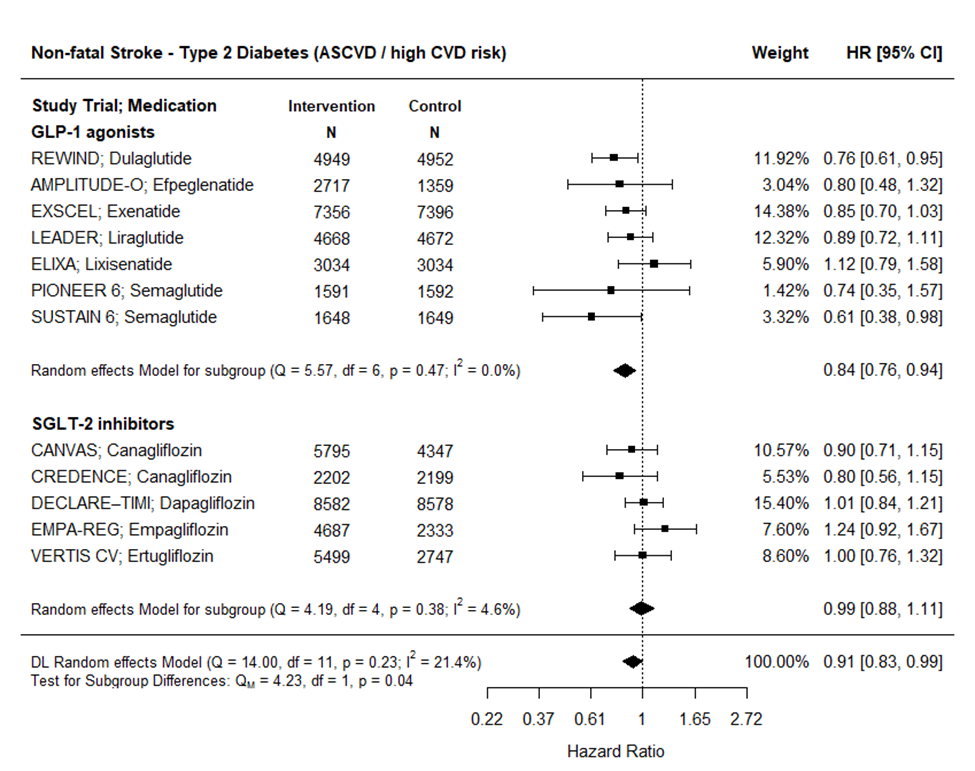


**Supplemental Figure S7 – Major Adverse Cardiac Events (MACE)
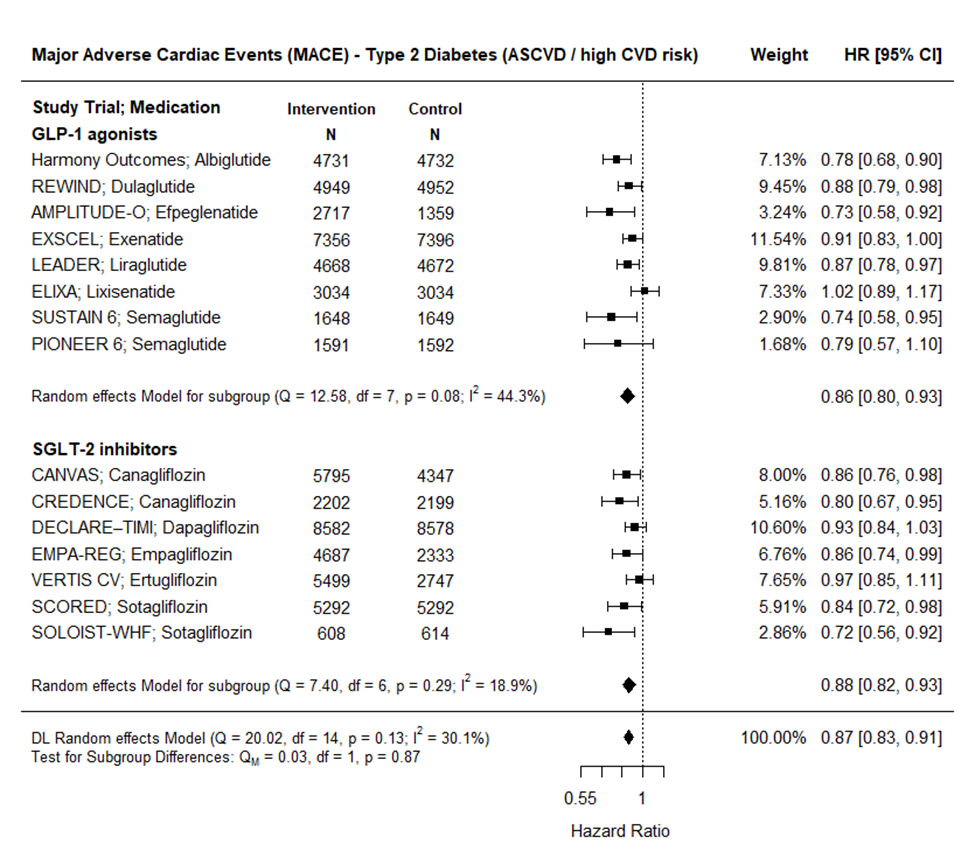
**

**Supplemental Figure S8 –** **Kidney Composite Outcomes**


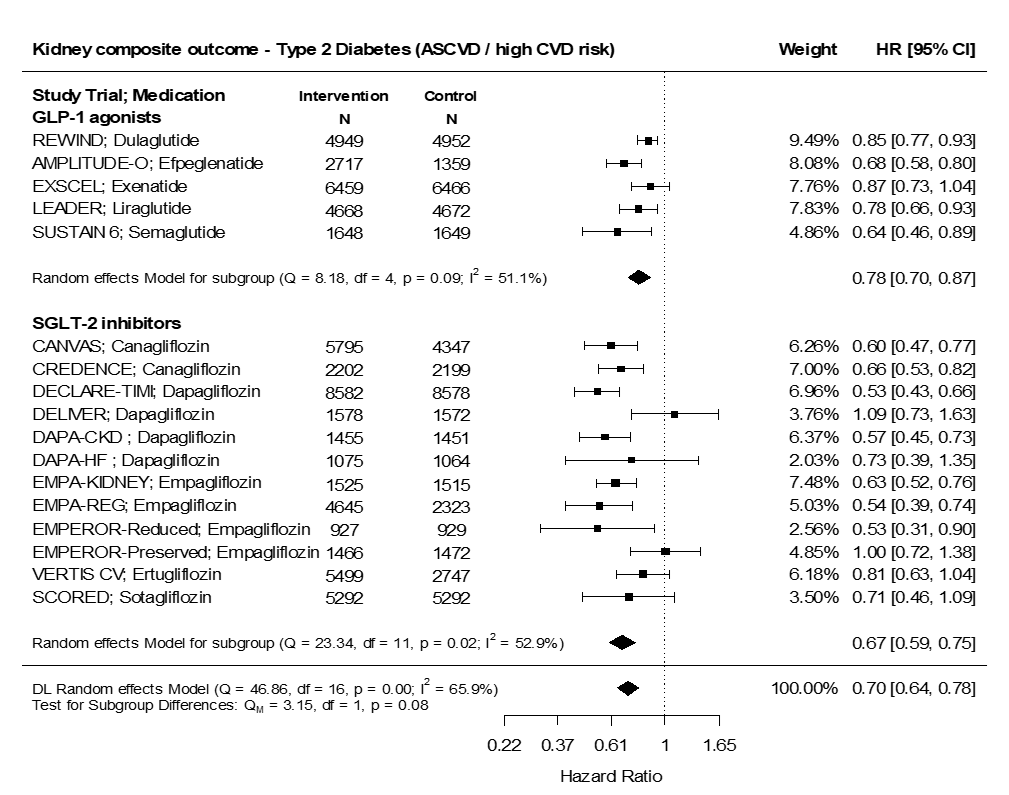


**Supplemental Figures S9 – S16: Meta-analysis of outcomes for people with Chronic Kidney Disease**

**Supplemental Figure S9 –** **Cardiovascular Mortality**


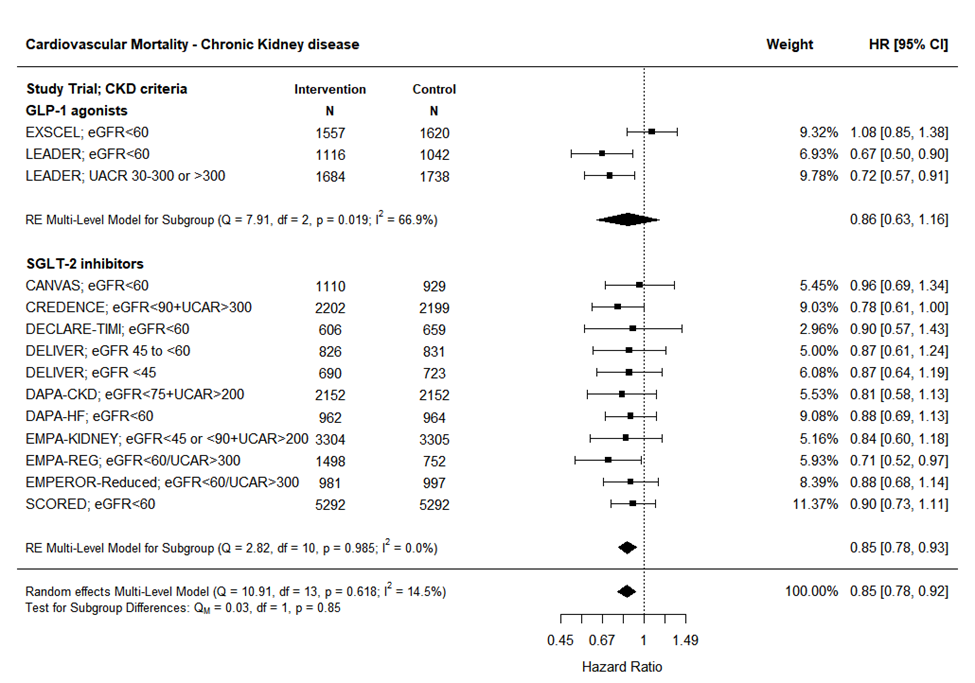


**Supplemental Figure S10 – Any-cause mortality**


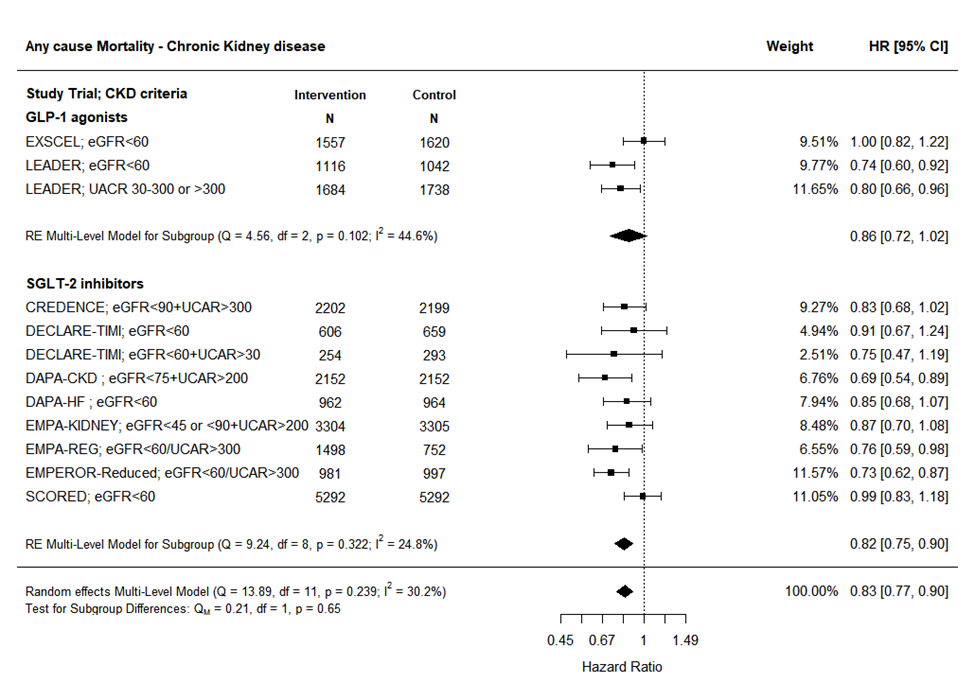


**Supplemental Figure S11 – Cardiovascular mortality or hospitalization due to heart failure**


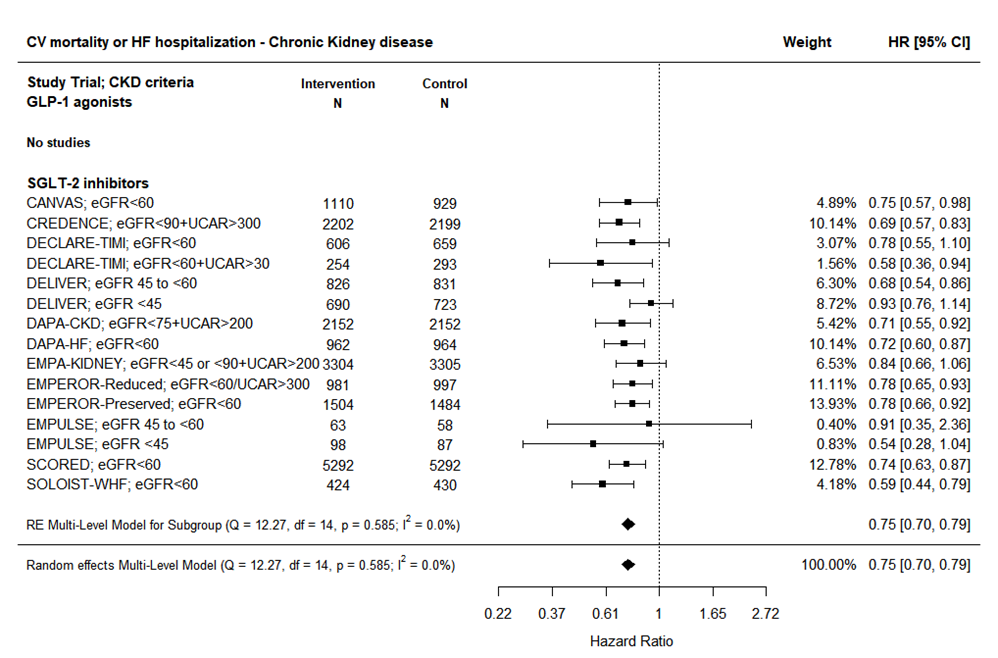


**Supplemental Figure S12 –** **Hospitalization due to heart failure**


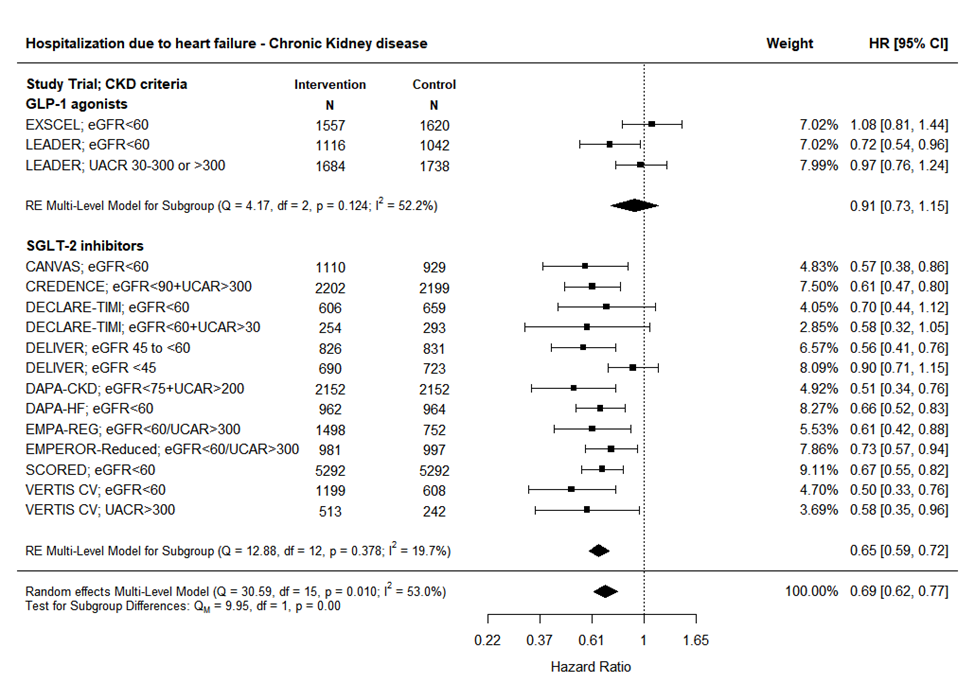


**Supplemental Figure S13 –** **Non-fatal myocardial infarction**


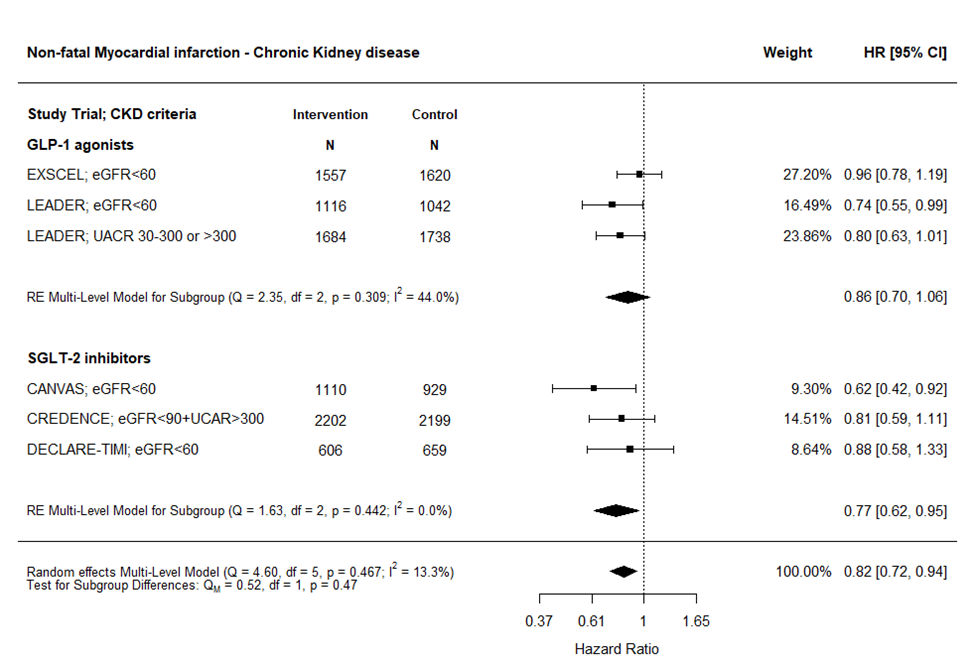


**Supplemental Figure S14 – Non-fatal stroke**


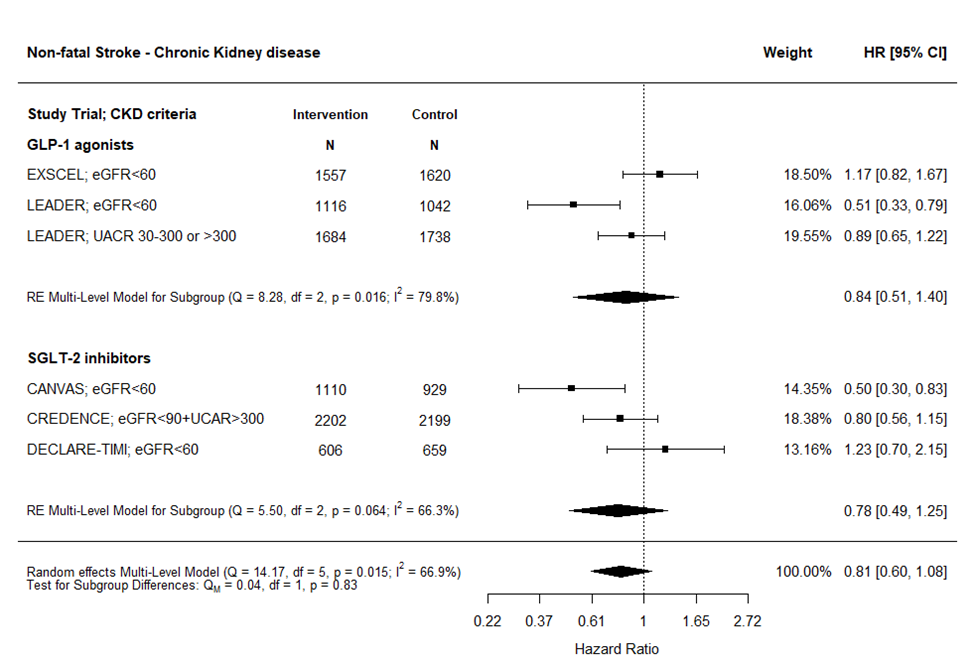


**Supplemental Figure S15 – Major Adverse Cardiac Events (MACE)**


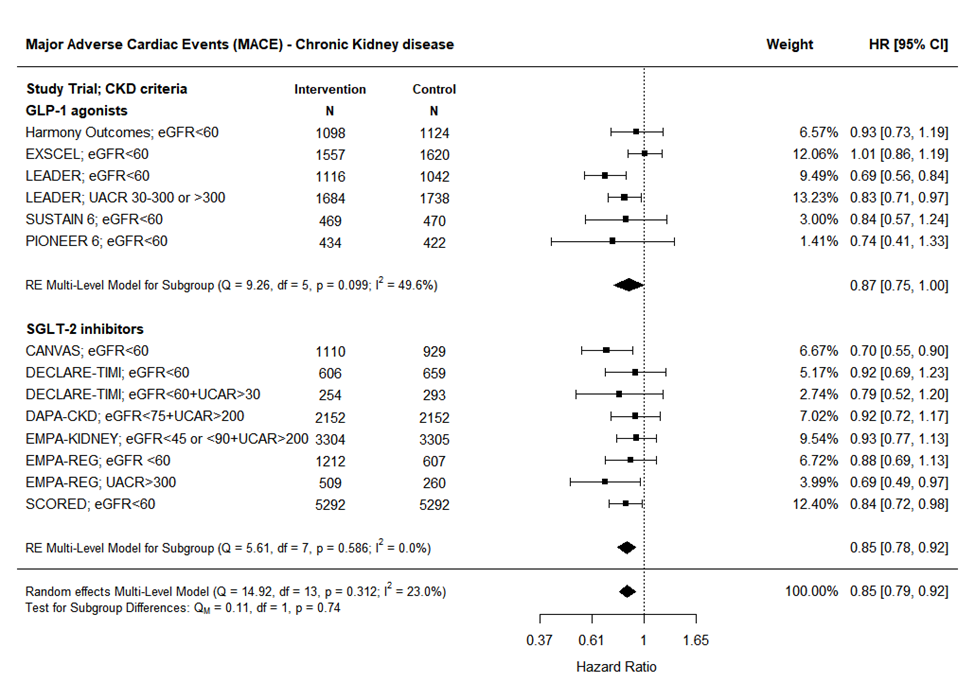


**Supplemental Figure S16 – Kidney Composite Outcome**


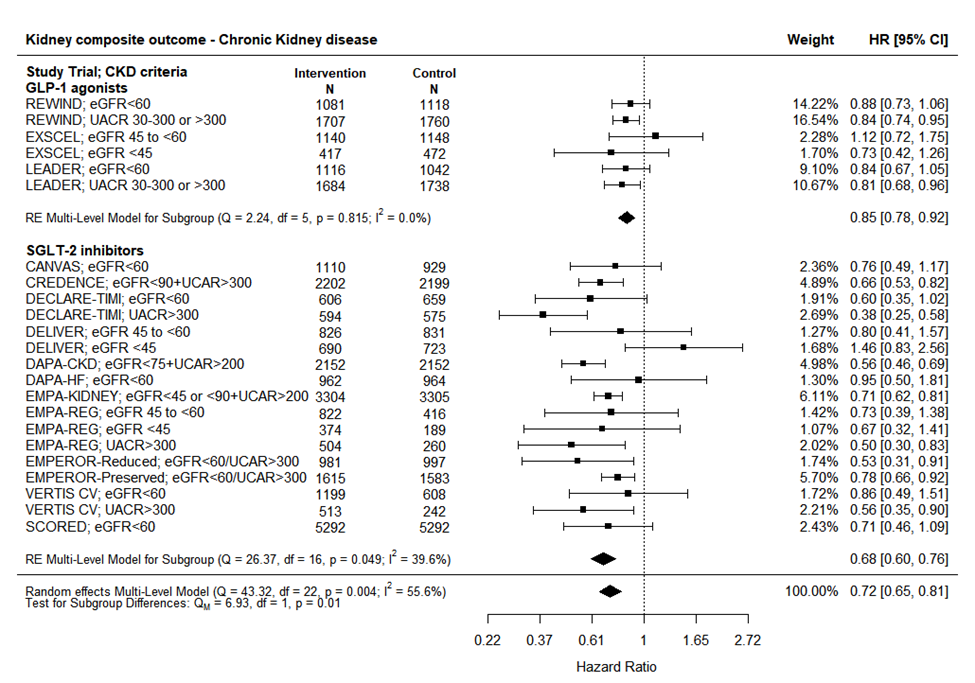


**Supplemental Figures S17 – S21: Meta-analysis for outcomes for people with Heart Failure**

**Supplemental Figure S17 – Cardiovascular mortality**


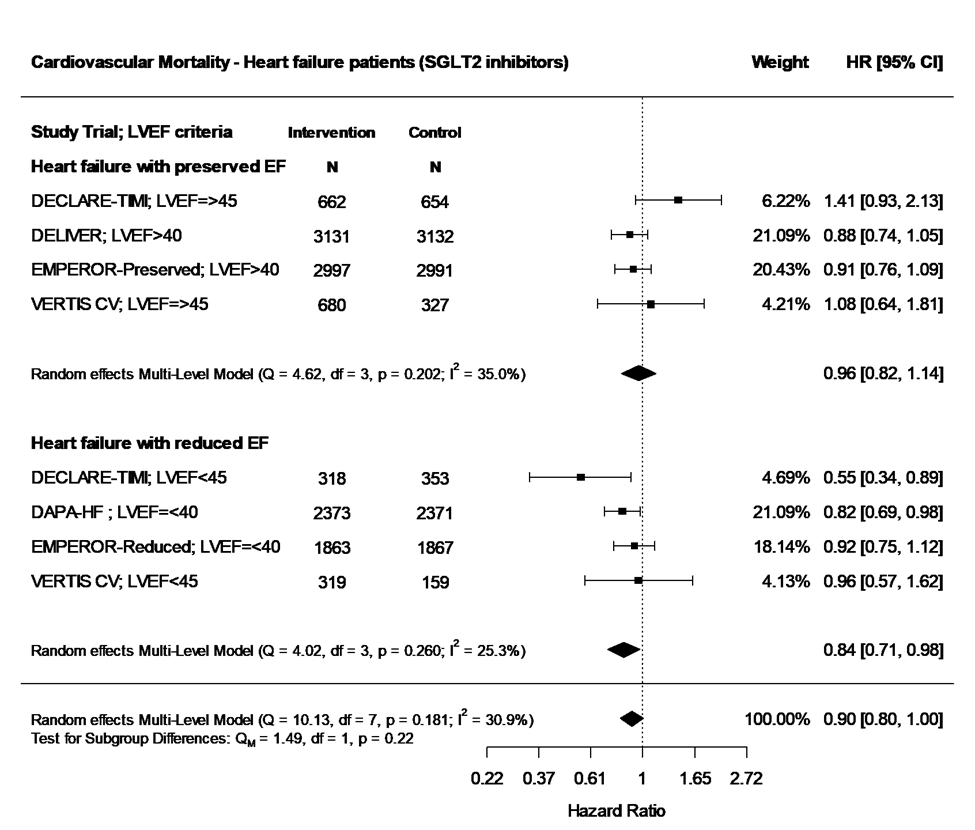


**Supplemental Figure S18 – Any-cause mortality**


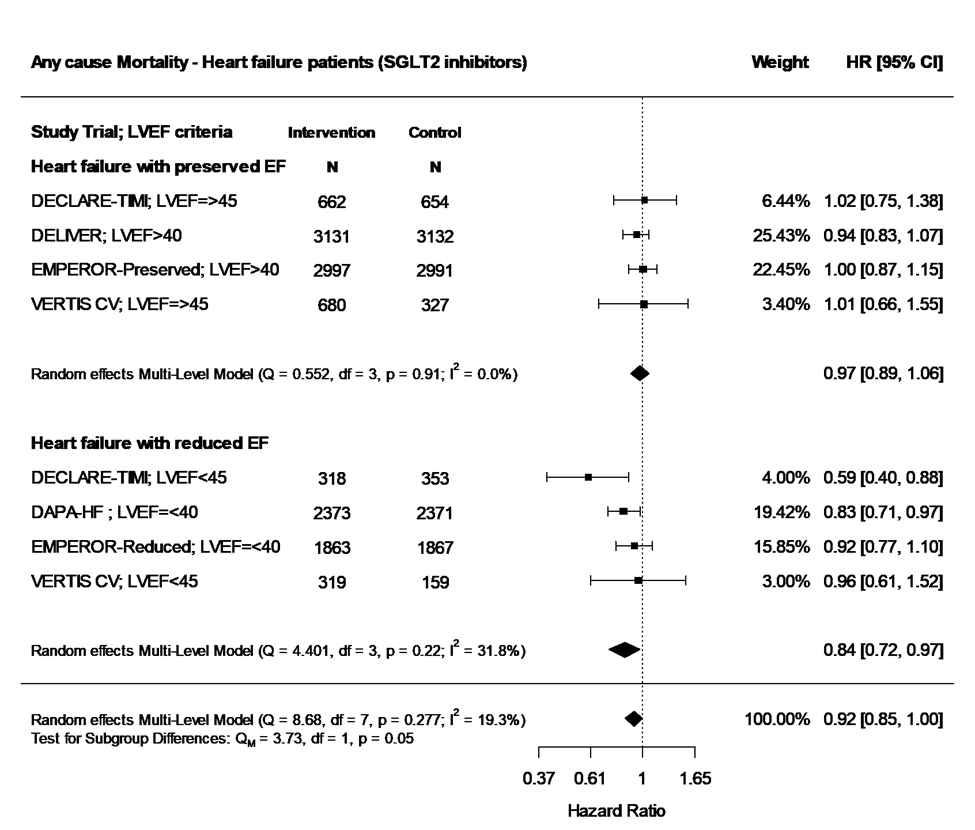


**Supplemental Figure S19 – Cardiovascular mortality or hospitalization due to heart failure**


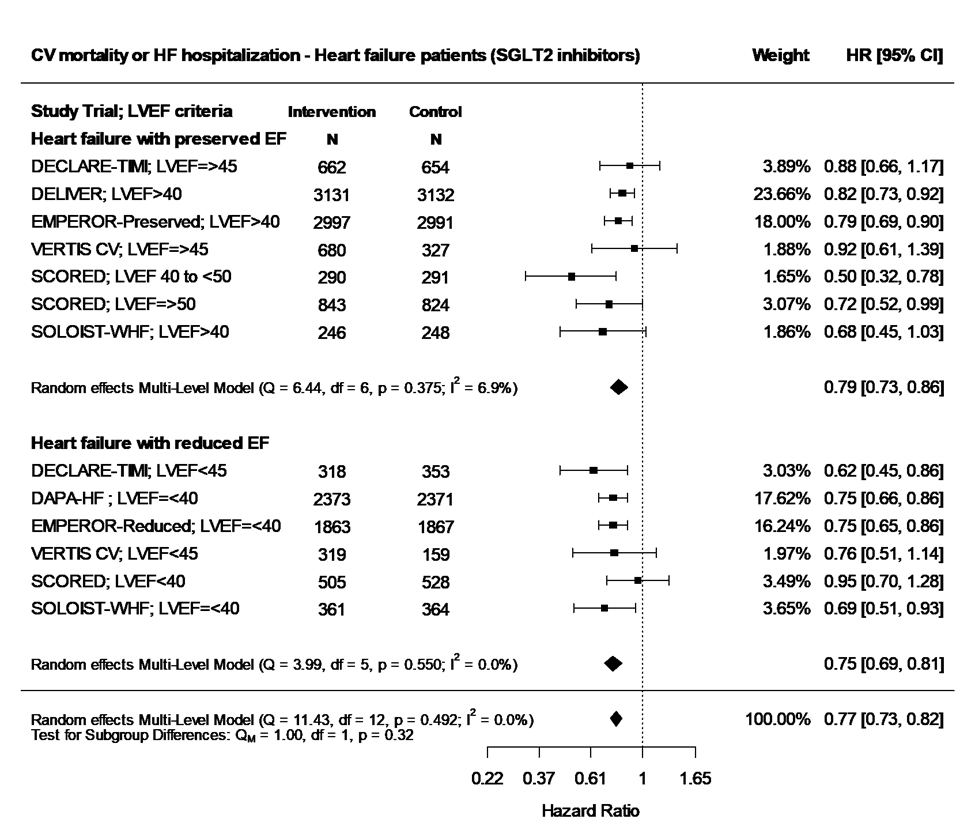


**Supplemental Figure S20 – Hospitalization due to heart failure**


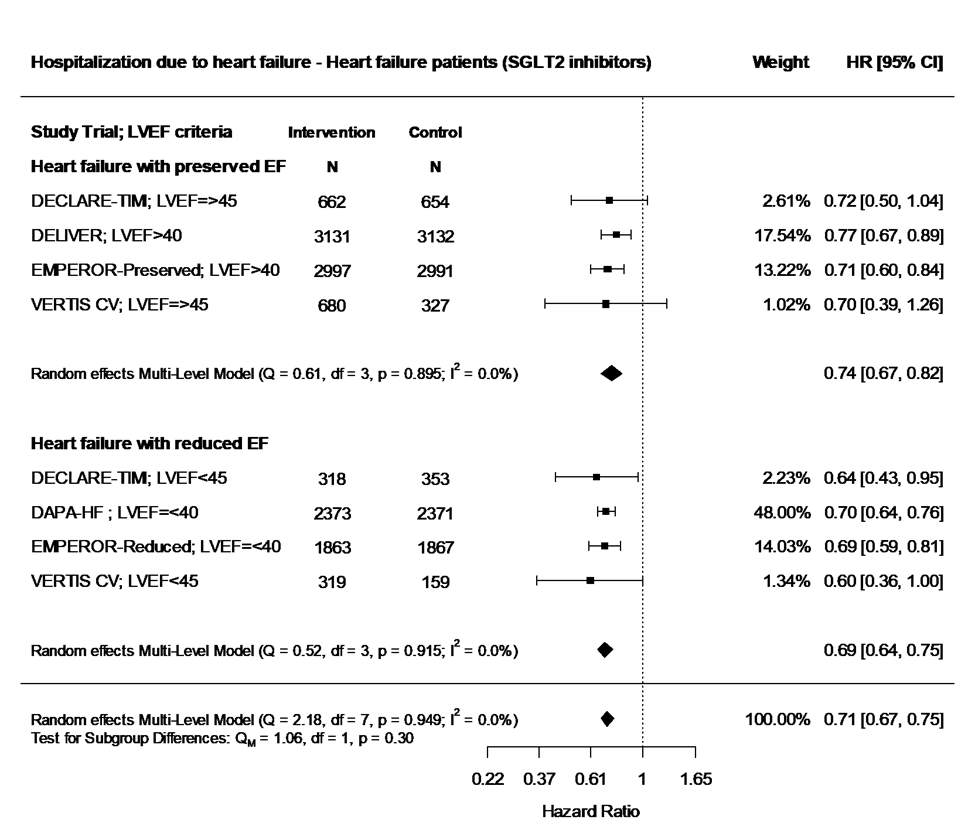


**Supplemental Figure S21 – Kidney Composite Outcome**


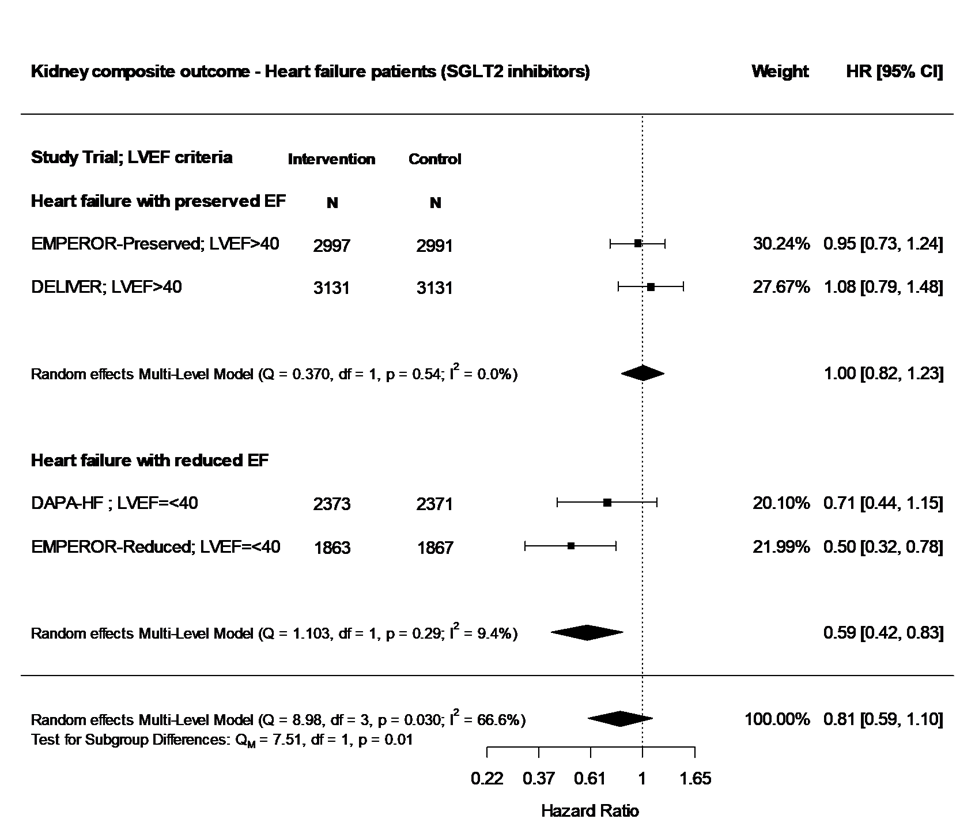


**Supplemental Table S1:** Characteristics of Included Studies for SGLT2i

| **Author, Year, Trial** | Bhatt, 2021, **SOLOIST-WHF** |
| --- | --- |
| **Question/Study Objective** | Hypothesized that sotagliflozin would reduce the risks of death from cardiovascular causes, hospitalization for heart failure, and an urgent visit for heart failure among patients with diabetes mellitus and recent worsening of heart failure with either reduced or preserved ejection fraction when administered soon after an episode of decompensated heart failure. |
| **Study Design** | Randomized controlled trial. |
| **Sample Selection** | Inclusion: 18 to 85 years of age and had been hospitalized because of the presence of signs and symptoms of heart failure and received treatment  with intravenous diuretic therapy; patients were also required to have received a previous diagnosis of type 2 diabetes before the index admission or to have laboratory evidence to support a diagnosis of type 2 diabetes during the index admission  Exclusion: end-stage heart failure or recent acute coronary syndrome; stroke;  percutaneous coronary intervention or coronary artery bypass surgery; or an estimated glomerular filtration rate of less than 30mL per minute per 1.73m^2^ of body surface area |
| **Baseline Age** | Median (interquartile range): I: 69 (63-76); C: 70 (64-76) |
| **Sample Size (N)** | I: 608; C: 614 |
| **Intervention** | Patients randomly assigned, either before or within three days after hospital discharge, to receive 200mg of sotagliflozin once daily (with a dose increase to 400 mg, depending on side effects). |
| **Control** | Placebo |
| **Primary and Secondary Outcome(s)** | Primary end point: deaths from cardiovascular causes and hospitalizations and urgent visits for heart failure;  Secondary: hospitalization for heart failure; death from cardiovascular causes; death by any cause; deaths from cardiovascular causes, hospitalizations for heart failure, nonfatal myocardial infarctions, and nonfatal strokes — total no. of events; deaths from cardiovascular causes, hospitalizations and urgent visits for heart failure, and events of heart failure during hospitalization |
| **Findings** | In patients with diabetes and recent worsening heart failure, sotagliflozin therapy, initiated before or shortly after discharge, resulted in a significantly lower total number of deaths from cardiovascular causes and hospitalizations and urgent visits for heart failure than placebo. |
| **Funding** | Sanofi (withdrawn) and Lexicon Pharmaceuticals |

| **Author, Year, Trial** | Heerspink, 2020, **DAPA-CKD** |
| --- | --- |
| **Question/Study Objective** | Assessed the long-term efficacy and safety of the Sodium–glucose-cotransporter 2 (SGLT2) inhibitor dapagliflozin in patients with chronic kidney disease, with or without type 2 diabetes. |
| **Study Design** | Randomized controlled trial. |
| **Sample Selection** | Inclusion: Adults with or without type 2 diabetes who had an estimated glomerular filtration rate (GFR) of 25 to 75mL per minute per 1.73m^2^ of body-surface area and a urinary albumin-to-creatinine ratio (with albumin measured in milligrams and creatinine measured in grams) of 200 to 5000; all the participants were required to be receiving a stable dose of an angiotensin-converting–enzyme (ACE) inhibitor or angiotensin-receptor blockers (ARBs) for at least four weeks before screening. However, participants who were documented to be unable to take ACE inhibitors or ARBs were allowed to participate  Exclusion: documented diagnosis of type 1 diabetes; polycystic kidney disease; lupus nephritis; or antineutrophil cytoplasmic antibody–associated vasculitis; received immunotherapy for primary or secondary kidney disease within six months before enrollment |
| **Baseline Age** | Mean (SD): I: 61.8 (12.1); C: 61.0 (12.1) |
| **Sample Size (N)** | I: 2152; C: 2152 |
| **Intervention** | Participants received dapagliflozin (10mg once daily). |
| **Control** | Placebo |
| **Primary and Secondary Outcome(s)** | The primary composite outcome, assessed in a time-to-event analysis, was the first occurrence of any of the following: a decline of at least 50% in the estimated GFR (confirmed by a second serum creatinine measurement after ≥28 days), the onset of end-stage kidney disease (defined as maintenance dialysis for ≥28 days, kidney transplantation, or an estimated GFR of <15mL per minute per 1.73m^2^ confirmed by a second measurement after ≥28 days), or death from renal or cardiovascular causes.  Secondary outcomes (also assessed in time-to-event analyses) were, in hierarchical order, the composite kidney outcome of a sustained decline in the estimated GFR of at least 50%, end-stage kidney disease, or death from renal causes; a composite cardiovascular outcome defined as hospitalization for heart failure or death from cardiovascular causes; and death from any cause. |
| **Findings** | Among patients with chronic kidney disease, regardless of the presence or absence of diabetes, the risk of a composite of a sustained decline in the estimated GFR of at least 50%, end-stage kidney disease, or death from renal or cardiovascular causes was significantly lower with dapagliflozin than with placebo. |
| **Funding** | AstraZeneca |

| **Author, Year, Trial** | Packer, 2020, **EMPEROR-Reduced** |
| --- | --- |
| **Question/Study Objective** | In the Empagliflozin Outcome Trial in Patients with Chronic Heart Failure and a Reduced Ejection Fraction (EMPEROR-Reduced), empagliflozin was evaluated in a population of patients with chronic heart failure and a reduced ejection fraction (with or without diabetes) that was enriched for patients with a greater severity of left ventricular systolic dysfunction. |
| **Study Design** | Randomized controlled trial. |
| **Sample Selection** | Inclusion: men or women, aged ≥ 18 years who have had chronic heart failure (functional class II, III or IV) for at least three months and whose left ventricular ejection fraction is ≤40% at its most recent assessment prior to enrolment Exclusion: cardiovascular diseases or treatments that increase the unpredictability of or change the patients' clinical course; independent of heart failure; significant co‐morbid conditions that might influence the clinical course; independent of heart failure; any condition that might jeopardize patient safety, limit the patients' participation in the trial, or undermine the interpretation of trial data |
| **Baseline Age** | I: 67.2(10.8); C: 66.5(11.2) |
| **Sample Size (N)** | I: 1863; C: 1867 |
| **Intervention** | Empagliflozin 10mg. |
| **Control** | Placebo |
| **Primary and Secondary Outcome(s)** | Primary: hospitalization from heart failure; cardiovascular death  Secondary: total number of hospitalizations for heart failure  Other: composite renal outcomes; change in quality of life; number of hospitalizations any cause; death by any cause; onset of new diabetes in patients with prediabetes and lab test results; body weight and blood pressure |
| **Findings** | Among patients receiving recommended therapy for heart failure, those in the empagliflozin group had a lower risk of cardiovascular death or hospitalization for heart failure than those in the placebo group, regardless of the presence or absence of diabetes. |
| **Funding** | Boehringer Ingelheim and Eli Lilly |

| **Author, Year, Trial** | McMurray, 2019, **DAPA-HF** |
| --- | --- |
| **Question/Study Objective** | The DAPA-HF (Dapagliflozin and Prevention of Adverse Outcomes in Heart Failure) trial evaluated the efficacy and safety of the SGLT2 inhibitor dapagliflozin in patients with heart failure and a reduced ejection fraction, regardless of the presence or absence of diabetes. |
| **Study Design** | Randomized controlled trial. |
| **Sample Selection** | Inclusion: at least 18 years, an ejection fraction of 40% or less; New York Heart Association (NYHA) class II, III, or IV symptoms; plasma level of N-terminal pro–B-type natriuretic peptide (NT-proBNP) of at least 600pg per mL (or ≥400pg per mL if they had been hospitalized for heart failure within the previous 12 months); patients with atrial fibrillation or atrial flutter on baseline electrocardiography were required to have an NT-proBNP level of at least  900pg per mL, regardless of their history of hospitalization for heart failure; received standard heart failure device therapy (an implantable cardioverter–defibrillator, cardiac resynchronization therapy, or both) and standard drug therapy, including an angiotensin-converting–enzyme inhibitor, an angiotensin-receptor blocker, or sacubitril–valsartan plus a beta-blocker, unless such use was contraindicated or resulted in unacceptable side effects (the use of a mineralocorticoid receptor antagonist was encouraged, drug doses were individually tailored, in accordance with guideline recommendations); patients with type 2 diabetes continued to take their glucose-lowering therapies, but doses could be adjusted as required (the dose of insulin and sulfonylurea could be reduced to minimize the risk of hypoglycemia (e.g., in patients with a glycated hemoglobin level of <7%))  Exclusion: recent treatment with or unacceptable side effects associated with  an SGLT2 inhibitor; type 1 diabetes mellitus; symptoms of hypotension or a systolic blood pressure of less than 95 mm Hg; and an estimated glomerular filtration rate (eGFR) below 30 mL per minute per 1.73m^2^ of body-surface area (or rapidly declining renal function) |
| **Baseline Age** | Mean (SD): 66.2(11.0); C 66.5(10.8) |
| **Sample Size (N)** | (I) 2373; (C) 2371 |
| **Intervention** | Dapagliflozin 10mg |
| **Control** | Placebo |
| **Primary and Secondary Outcome(s)** | Primary: a composite of worsening heart failure or death from cardiovascular causes  Secondary outcomes: a composite of hospitalization for heart failure or cardiovascular death. Total number of hospitalizations for heart failure (including repeat admissions) and cardiovascular deaths; the change from baseline to eight months in the total symptom score on the Kansas City Cardiomyopathy Questionnaire, a composite of worsening renal function, or renal death; and death from any cause |
| **Findings** | Among patients with heart failure and a reduced ejection fraction, the risk of worsening heart failure or death from cardiovascular causes was lower among those who received dapagliflozin than among those who received placebo, regardless of the presence or absence of diabetes. |
| **Funding** | AstraZeneca |

| **Author, Year, Trial** | Wivott, 2019, **DECLARE–TIMI** |
| --- | --- |
| **Question/Study Objective** | The Dapagliflozin Effect on Cardiovascular Events–Thrombolysis in Myocardial Infarction 58 (DECLARE–TIMI 58) trial evaluated the effects of dapagliflozin on cardiovascular and renal outcomes in a broad population of patients who had or were at risk for atherosclerotic cardiovascular disease. |
| **Study Design** | Randomized controlled trial. |
| **Sample Selection** | Inclusion: ≥40 years and had type 2 diabetes; a glycated hemoglobin level of at least 6.5% but less than 12.0%; and a creatinine clearance of 60mL or more per minute; multiple risk factors for atherosclerotic cardiovascular disease or had established atherosclerotic cardiovascular disease (defined as clinically evident ischemic heart disease, ischemic cerebrovascular disease, or peripheral artery disease). Participants with multiple risk factors were men 55 years of age or older or women 60 years of age or older who had one or more traditional risk factors, including hypertension, dyslipidemia (defined as a low-density lipoprotein cholesterol level >130mg per dL [3.36mmol per L] or the use of lipid-lowering therapies), or use of tobacco  Exclusion: NR |
| **Baseline Age** | Mean (SD): I: 63.9 (6.8); C: 64.0 (6.8) |
| **Sample Size (N)** | I: 8582; C: 8578 |
| **Intervention** | Dapagliflozin 10mg |
| **Control** | Placebo |
| **Primary and Secondary Outcome(s)** | Primary: MACE (defined as cardiovascular death, myocardial infarction, or  ischemic stroke). The two primary efficacy outcomes were MACE and a composite of cardiovascular death or hospitalization for heart failure.  Secondary: renal composite outcome, death by any cause |
| **Findings** | In patients with type 2 diabetes who had or were at risk for atherosclerotic cardiovascular disease, treatment with dapagliflozin did not result in a higher or lower rate of MACE than placebo but did result in a lower rate of cardiovascular death or hospitalization for heart failure, a finding that reflects a lower rate of hospitalization for heart failure. |
| **Funding** | AstraZeneca |

| **Author, Year, Trial** | Perkovic, 2019, **CREDENCE** |
| --- | --- |
| **Question/Study Objective** | The CREDENCE (Canagliflozin and Renal Events in Diabetes with Established Nephropathy Clinical Evaluation) trial assessed the effects of the SGLT2 inhibitor canagliflozin on renal outcomes in patients with type 2 diabetes and albuminuric chronic kidney disease. |
| **Study Design** | Randomized controlled trial. |
| **Sample Selection** | Inclusion: at least 30 years of age and had type 2 diabetes; with a glycated hemoglobin level of 6.5 to 12.0% (6.5 to 10.5% in Germany, according to a country amendment) also required to have chronic kidney disease Exclusion: had suspected nondiabetic kidney disease or type 1 diabetes; treated with immunosuppression for kidney disease; or had a history of dialysis or kidney transplantation |
| **Baseline Age** | Mean (SD): I: 63.9(9.2); C: 63.2 (9.2) |
| **Sample Size (N)** | I: 2202; C: 2199 |
| **Intervention** | Canagliflozin 100mg |
| **Control** | Placebo |
| **Primary and Secondary Outcome(s)** | Primary: doubling of serum creatinine level, end-stage kidney disease, renal death, cardiovascular death  Secondary: cardiovascular death, death by any cause, hospitalization from heart failure |
| **Findings** | In patients with type 2 diabetes and kidney disease, the risk of kidney failure and cardiovascular events was lower in the canagliflozin group than in the placebo group at a median follow-up of 2.62 years. |
| **Funding** | Janssen Research and Development |

| **Author, Year, Trial** | Neal, 2017, **CANVAS** |
| --- | --- |
| **Question/Study Objective** | Reported the effects of treatment with canagliflozin on cardiovascular, renal, and safety outcomes for participants with type 2 diabetes and high cardiovascular risk. |
| **Study Design** | Randomized controlled trial. |
| **Sample Selection** | Inclusion: identical in the two trials; participants were men and women with type 2 diabetes (glycated hemoglobin level, ≥7.0% and ≤10.5%) and were either 30 years of age or older with a history of symptomatic atherosclerotic cardiovascular disease or 50 years of age or older with two or more of the following risk factors for cardiovascular disease: duration of diabetes of at least 10 years, systolic blood pressure higher than 140 mmHg while they were  receiving one or more antihypertensive agents, current smoking, microalbuminuria or macroalbuminuria, or high-density lipoprotein (HDL) cholesterol level of less than 1mmol per liter (38.7mg per deciliter); estimated glomerular filtration rate (eGFR) at entry of more than 30mL per minute per 1.73m^2^ of body-surface area and to meet a range of other criteria  Exclusion: NR |
| **Baseline Age** | Mean (SD) I: 63.2 (8.3); C: 63.4 (8.2) |
| **Sample Size (N)** | I: 5795; C: 4347 |
| **Intervention** | Received canagliflozin at a dose of 100 or 300 mg and randomly assigned in a  1:1 ratio to receive canagliflozin, administered at an initial dose of 100 mg daily with an optional increase to 300 mg starting from week 13. |
| **Control** | Placebo |
| **Primary and Secondary Outcome(s)** | Primary: composite of death from cardiovascular causes, nonfatal myocardial infarction, or nonfatal stroke  Secondary: planned for sequential conditional hypothesis testing were death from any cause, death from cardiovascular causes, progression of albuminuria, and the composite of death from cardiovascular causes and hospitalization for heart failure  Exploratory cardiovascular outcomes: nonfatal myocardial infarction, nonfatal stroke, and hospitalization for heart failure, and the key prespecified exploratory renal outcomes were regression of albuminuria (using criteria comparable to those defined for category progression) and the renal composite comprising a 40% reduction in eGFR sustained for at least two consecutive measures, the need for renal-replacement therapy (dialysis or transplantation), or death from renal causes (defined as death with a proximate renal cause). Evaluation of total hospitalizations was also prespecified.  Adverse events |
| **Findings** | In two trials involving patients with type 2 diabetes and an elevated risk of cardiovascular disease, patients treated with canagliflozin had a lower risk of cardiovascular events than those who received placebo but a greater risk of amputation, primarily at the level of the toe or metatarsal. |
| **Funding** | Janssen Research and Development |

| **Author, Year, Trial** | Zinman, 2015, **EMPA-REG OUTCOME** |
| --- | --- |
| **Question/Study Objective** | Examined the effects of empagliflozin, as compared with placebo, on cardiovascular morbidity and mortality in patients with type 2 diabetes at high risk for cardiovascular events who were receiving standard care. |
| **Study Design** | Randomized controlled trial. |
| **Sample Selection** | Inclusion: type 2 diabetes were adults (≥18 years of age); body-mass index of 45 or less and an estimated glomerular filtration rate (eGFR) of at least 30mL per minute per 1.73 m^2^ of body-surface area; according to the Modification of Diet in Renal Disease criteria; established cardiovascular disease; received  no glucose-lowering agents for at least 12 weeks before randomization; glycated hemoglobin level of at least 7.0% and no more than 9.0% or had received stable glucose-lowering therapy for at least 12 weeks before randomization; glycated hemoglobin level of at least 7.0% and no more than 10.0%  Exclusion: Uncontrolled hyperglycemia with glucose >240 mg/dL after an overnight fast during placebo run-in and confirmed by a second measurement (not on the same day); indication of liver disease, defined by serum levels of alanine amininotransferase, aspartate aminotransferase, or alkaline phosphatase above three times the upper limit of normal during screening or run-in phase; planned cardiac surgery or angioplasty within three months; estimated glomerular filtration rate <30 mL/min/1.73m^2^ (according to the Modification of Diet in Renal Disease equation) at screening or during run-in phase; bariatric surgery within the past two years and other gastrointestinal surgeries that induced chronic malabsorption; blood dyscrasias or any disorders causing hemolysis or unstable red blood cells; medical history of cancer (except for basal cell carcinoma) and/or treatment for cancer within the last five years; contraindications to background therapy according to the local label; treatment with anti-obesity drugs three months prior to informed consent or any other  treatment at time of screening leading to unstable body weight; treatment with systemic steroids at time of informed consent or change in dosage of thyroid hormones within six weeks prior to informed consent; any uncontrolled endocrine disorder except type 2 diabetes; pre-menopausal women (last menstruation ≤one year prior to informed consent) who were nursing, pregnant, or of child-bearing potential and were not practicing an acceptable  method of birth control, or did not plan to continue using this method throughout the study, or did not agree to submit to periodic pregnancy testing during the trial (acceptable methods of birth control include tubal ligation, transdermal patch, intrauterine devices/systems, oral, implantable or injectable contraceptives, sexual abstinence, double barrier method, vasectomy of partner); alcohol or drug abuse within three months of informed consent that would interfere with trial participation or any ongoing condition leading to decreased compliance with study procedures or study drug intake; intake of an investigational drug in another trial within 30 days prior to intake of study  medication in this trial or participating in another trial involving an investigational drug and/or follow-up; any clinical condition that would jeopardize patient safety while participating in this clinical trial (in Canada, this included current genito-urinal infection or genito-urinal infection within two weeks prior to informed consent); acute coronary syndrome, stroke, or transient ischemic attack within two months prior to informed consent; in South Africa: blood pressure >160/100 mmHg at screening |
| **Baseline Age** | <65 years, N: I: 2596; C: 1297  ≥65 years, N: I: 2091; C: 1036 |
| **Sample Size (N)** | I: 4687; C: 2333 |
| **Intervention** | Received either 10 or 25mg of empagliflozin once daily. Background glucose-lowering therapy was to remain unchanged for the first 12 weeks after  randomization, although intensification was permitted if the patient had a confirmed fasting glucose level of more than 240mg per deciliter (>13.3mmol per liter). In cases of medical necessity, dose reduction or discontinuation of background medication could occur. After week 12, investigators were encouraged to adjust glucose lowering therapy at their discretion to achieve  glycemic control according to local guidelines. |
| **Control** | Placebo |
| **Primary and Secondary Outcome(s)** | Primary: composite of death from cardiovascular causes, nonfatal myocardial infarction (excluding silent myocardial infarction), or nonfatal stroke  Secondary: composite of the primary outcome plus hospitalization for unstable angina  Safety was assessed on the basis of adverse events that occurred during treatment or within seven days after the last dose of a study drug and were coded with the use of the Medical Dictionary for Regulatory Activities, version 18.0  Adverse events: hypoglycemic adverse events (plasma glucose level, ≤70 mg per deciliter [3.9 mmol per liter] or an event requiring assistance), and adverse events reflecting urinary tract infection, genital infection, volume depletion, acute renal failure, bone fracture, diabetic ketoacidosis, and thromboembolic events |
| **Findings** | Patients with type 2 diabetes at high risk for cardiovascular events who received empagliflozin, as compared with placebo, had a lower rate of the primary composite cardiovascular outcome and of death from any cause when the study drug was added to standard care. |
| **Funding** | Boehringer Ingelheim and Eli Lilly |

| **Author, Year, Trial** | Cannon, 2020, **VERTIS CV** |
| --- | --- |
| **Question/Study Objective** | The long-term effects of ertugliflozin on cardiovascular and renal outcomes were assessed in the Evaluation of Ertugliflozin Efficacy and Safety Cardiovascular Outcomes Trial (VERTIS CV). |
| **Study Design** | Randomized controlled trial |
| **Sample Selection** | Inclusion: ≥40 years; type 2 diabetes mellitus (T2DM) in accordance with American Diabetes Association (ADA) guidelines; HbA1c of 7.0–10.5% (53–91 mmol/mol) at the Screening visit (V1) on stable allowable anti-hyperglycemic agent(s) (AHA) or on no background AHA for at least eight weeks prior to V1; Body mass index ≥18.0 kg/m^2^; atherosclerosis involving the coronary, cerebral, or peripheral vascular systems  Exclusion: Patients who had been previously randomized into this trial; experienced a cardiovascular event (e.g., MI or stroke) or undergone coronary  angioplasty or peripheral intervention procedure between V1 and randomization; undergone any cardiovascular surgery within three months of V1; New York Heart Association Class IV heart failure (HF) at V1; Mean value for triplicate screening sitting systolic blood pressure (SBP) >160mmHg and/or  diastolic blood pressure (DBP) >90mmHg after at least a five-minute seated rest; clinically significant electrocardiogram (ECG) abnormality; history of type 1 diabetes mellitus, ketoacidosis or other specific types of diabetes; active, obstructive uropathy or indwelling urinary catheter; malignancy ≤five years prior to signing informed consent, except for adequately treated basal cell or squamous cell skin cancer or in situ cervical cancer; routinely consumes >two alcoholic drinks per day or >14 alcoholic drinks per week, or engages in binge drinking; clinically significant malabsorption condition; known hypersensitivity or intolerance to any sodium-glucose co-transporter-2 inhibitor; screening fasting plasma or finger-stick glucose >270 mg/dl (15 mmol/l); one or more severe hypoglycemic episodes within six months of V1; fasting triglycerides >600 mg/dl (6.78 mmol/L); BP or lipid altering medications; any of the following four categories: weight-loss program and is not weight-stable, weight-loss medication (e.g., orlistat, phentermine/topiramate, lorcaserin) and is not weight-stable, other medications associated with weight changes (e.g., anti-psychotic agents) and is not weight-stable, undergone bariatric surgery >12 months prior to V1 (or within 12 months of V1) and is not weight-stable (weight-stable is defined as <5% change in body weight in the last six months); treated for hyperthyroidism; estimated glomerular filtration rate <30 mL/min/1.73 m^2^; hemoglobin <10 g/dL (100 g/L); aspartate aminotransferase or alanine aminotransferase >two times the upper limit of normal (ULN) or a total bilirubin >1.5 X the ULN unless the patient has a history of Gilbert’s syndrome; medical history of active liver disease (other than non-alcoholic hepatic steatosis); is on or likely to require treatment for ≥14 consecutive days or repeated courses of pharmacologic doses of corticosteroids; the following therapeutic agents were prohibited for the duration of the trial: treatment with another SGLT2 inhibitor, rosiglitazone and chlorpropamide; donated blood or blood products within six weeks of V1; undergone a surgical procedure within four weeks prior to signing informed consent or have planned major surgery during the trial; known history of Human Immunodeficiency Virus; blood dyscrasias or any disorders causing hemolysis or unstable red blood cells; other severe acute or chronic medical or psychiatric condition or laboratory abnormality; previously been randomized in a trial with ertugliflozin; participation in other studies involving investigational drug(s) (phases 1–4) within 30 days before V1; pregnant or breast-feeding, or was expected to conceive during the trial; expected to undergo hormonal therapy in preparation to donate eggs during the period of the trial; patients who were investigational site staff members directly involved in the conduct of the trial and their family members, site staff members otherwise supervised by the investigator, or  patients who were Pfizer/Merck employees directly involved in the conduct of the trial |
| **Baseline Age** | Mean (SD): I: 64.4 (8.1); C: 64.4 (8.0) |
| **Sample Size (N)** | I: 5499; C: 2747 |
| **Intervention** | 5 or 15mg of ertugliflozin once daily. |
| **Control** | Placebo |
| **Primary and Secondary Outcome(s)** | Primary: composite of death from cardiovascular causes, nonfatal myocardial infarction, or nonfatal stroke (i.e., a major adverse cardiovascular event).  Secondary: composite of death from cardiovascular causes or hospitalization for heart failure; death from cardiovascular causes; and a composite of death from renal causes, renal replacement therapy, or doubling of the serum creatinine level  Adverse outcomes |
| **Findings** | Among patients with type 2 diabetes and atherosclerotic cardiovascular disease, ertugliflozin was noninferior to placebo with respect to major adverse cardiovascular events. |
| **Funding** | Merck Sharp & Dohme and Pfizer |

| **Author, Year, Trial** | Bhatt, 2020, **SCORED** |
| --- | --- |
| **Question/Study Objective** | Determined whether sotagliflozin was noninferior to placebo with respect to ischemic events and whether it was superior with respect to heart failure events. |
| **Study Design** | Randomized controlled trial. |
| **Sample Selection** | Inclusion: ≥18 years with type 2 diabetes mellitus with a glycated hemoglobin level of 7% or higher, chronic kidney disease (estimated glomerular filtration rate (eGFR) 25 to 60mL per minute per 1.73m^2^ of body-surface area), and additional cardiovascular risk factors. The risk factors consisted of at least one major cardiovascular risk factor in those ≥18 years or at least two minor cardiovascular risk factors in those ≥55 years  Exclusion: any plan to start an SGLT2 inhibitor during the trial |
| **Baseline Age** | Median (Interquartile range) I: 69 (63–74); C: 69 (63–74) |
| **Sample Size (N)** | I: 5292; C: 5292 |
| **Intervention** | Sotagliflozin, 200mg once daily, with an increase to 400mg once daily if unacceptable side effects did not occur. |
| **Control** | Placebo |
| **Primary and Secondary Outcome(s)** | Primary: total number of deaths from cardiovascular causes, hospitalizations for heart failure, and urgent visits for heart failure  Secondary: total number of hospitalizations for heart failure and urgent visits for heart failure; deaths from cardiovascular causes; the total number of deaths from cardiovascular causes, hospitalizations for heart failure, nonfatal myocardial infarctions, and nonfatal strokes; the total number of deaths from cardiovascular causes, hospitalizations for heart failure, urgent visits for heart failure, and events of heart failure during hospitalization; the first occurrence of the composite of a sustained decrease of at least 50% in the eGFR from baseline for at least 30 days, long-term dialysis, renal transplantation, or a sustained eGFR of less than 15mL per minute per 1.73m^2^ for at least 30 days; deaths from any cause; and the total number of deaths from cardiovascular causes, nonfatal myocardial infarctions, and nonfatal strokes |
| **Findings** | In patients with diabetes and chronic kidney disease, with or without albuminuria, sotagliflozin resulted in a lower risk of the composite of deaths from cardiovascular causes, hospitalizations for heart failure, and urgent visits for heart failure than placebo but was associated with adverse events. |
| **Funding** | Sanofi and Lexicon Pharmaceuticals |

| **Author, Year, Trial** | Herrington, 2022**, EMPA-KIDNEY** |
| --- | --- |
| **Question/Study Objective** | Assessed the effect of once-daily empagliflozin treatment on the progression of kidney disease and cardiovascular disease and examined the safety profile of the drug in a wide range of patients with chronic kidney disease. |
| **Study Design** | Randomized clinical trial. |
| **Sample Selection** | Inclusion: adults with a race-adjusted eGFR (calculated with the use of the Chronic Kidney Disease Epidemiology Collaboration formula) of at least 20 but less than 45mL per minute per 1.73m^2^, regardless of the level of albuminuria, or with an eGFR of at least 45 but less than 90 mL per minute per 1.73m^2^ with a urinary albumin-to-creatinine ratio of at least 200 at the screening visit; required to be taking a clinically appropriate dose of a single-agent renin–angiotensin system (RAS) inhibitor, but patients could be included, as specified in the protocol, if an investigator judged that a RAS inhibitor was not indicated or would not be not tolerated; patients with or without diabetes.    Exclusion: with polycystic kidney disease and those who had received a kidney transplant. |
| **Baseline Age** | O: 63.8; I: 63.9 (13.9); C: 63.8 (13.9) |
| **Sample Size (N)** | O: 6609; I: 3304; C: 3305 |
| **Intervention** | All eligible patients entered a pre-randomization run-in phase and were provided with a 15-week supply of once-daily placebo tablets. Received empagliflozin (10 mg once daily). |
| **Control** | Placebo. |
| **Primary and Secondary Outcome(s)** | Primary: first occurrence of progression of kidney disease or death from cardiovascular causes. Progression of kidney disease was defined as end-stage kidney disease (ESKD; the initiation of maintenance dialysis or receipt of a kidney transplant), a sustained decrease in the eGFR to less than 10 ml per minute per 1.73 m2, a sustained decrease from baseline in the eGFR of at least 40%, or death from renal causes.    Secondary: a composite of hospitalization for heart failure or death from cardiovascular causes, hospitalization for any cause (including the first and any subsequent hospitalizations), and death from any cause, progression of kidney disease, death from cardiovascular causes, and a composite of ESKD or  death from cardiovascular causes. |
| **Findings** | Among a wide range of patients with chronic kidney disease who were at risk for disease progression, empagliflozin therapy led to a lower risk of progression of kidney disease or death from cardiovascular causes than placebo. |
| **Funding** | Boehringer Ingelheim and and Eli Lilly, United Kingdom Medical Research Council (MRC), the British Heart Foundation, National Institute for Health and Care Research Biomedical Research Council, and Health Data Research UK. |

| **Author, Year, Trial** | Solomon, 2022, **DELIVER** |
| --- | --- |
| **Question/Study Objective** | Tested the hypothesis that the SGLT2 inhibitor dapagliflozin would reduce the risk of worsening heart failure or cardiovascular death among patients with a mildly reduced or preserved ejection fraction. |
| **Study Design** | Randomized controlled trial. |
| **Sample Selection** | Inclusion: Ability to give written informed consent; men and women age ≥ 40 years; documented diagnosis of symptomatic heart failure (New York Heart Association [NYHA] class II-IV) at enrollment, and a medical history of typical symptoms/signs of heart failure ≥ 6 weeks before enrollment with at least intermittent need for diuretic treatment (requiring recurrent intermittent dosing); Left Ventricular Ejection Fraction (LVEF) > 40% and evidence of structural heart disease (i.e. left ventricular hypertrophy or left atrial enlargement) documented by the most recent echocardiogram, and/or cardiac MRI within the last 12 months prior to enrollment. For patients with prior acute cardiac events or procedures that may reduce LVEF qualifying cardiac imaging assessment at least 12 weeks following the procedure/event is required. Structural heart disease was defined as:  • Left atrial (LA) enlargement with at least one of the following: LA width (diameter) ≥3.8cm or LA length ≥5.0cm, or LA area ≥20cm, or LA volume ≥55mL or LA volume index ≥29mL/m.  • Left ventricular hypertrophy with septal thickness or posterior wall thickness ≥1.1cm;  NT-pro BNP ≥300pg/mL at Visit 1 for patients without ongoing atrial fibrillation/flutter. If ongoing atrial fibrillation/flutter at Visit 1, NT-pro BNP must be ≥600pg/mL; Patients may be ambulatory, or hospitalized; patients must be off intravenous heart failure therapy (including diuretics) for at least 12 hours prior to enrollment and 24 hours prior to randomization.    Exclusion: therapy with an SGLT2 inhibitor within 4 weeks prior to randomization or previous intolerance to an SGLT2 inhibitor; Type 1 diabetes mellitus; eGFR <25mL/min/1.73m^2^ (CKD-EPI formula) at Visit 1; systolic blood pressure (BP) <95mmHg on 2 consecutive measurements at 5-minute intervals, at Visit 1 or at Visit 2; systolic BP ≥160mmHg if not on treatment with ≥ 3 blood pressure lowering medications or ≥180mmHg irrespective of treatments, on 2 consecutive measurements at 5-minute intervals, at Visit 1 or at Visit 2; myocardial infarction (MI), unstable angina, coronary revascularization (percutaneous coronary intervention (PCI) or coronary artery bypass grafting (CABG)), ablation of atrial flutter/fibrillation, valve repair/replacement within 12 weeks prior to enrollment. Before enrollment, these patients must have their qualifying echocardiography and/or cardiac MRI examination at least 12 weeks after the event; planned coronary revascularization, ablation of atrial flutter/fibrillation and valve repair/replacement; stroke or transient ischemic attack within 12 weeks prior to enrollment; probable alternative or concomitant diagnoses which in the opinion of the investigator could account for the patient's heart failure symptoms and signs (e.g. anemia, hypothyroidism); Body mass index >50 kg/m^2^; World Health Organization Group 1 pulmonary hypertension, chronic pulmonary embolism, severe pulmonary disease, including chronic obstructive pulmonary disease (i.e., requiring home oxygen, chronic nebulizer therapy or chronic oral steroid therapy, or hospitalization for exacerbation of chronic obstructive pulmonary disease requiring ventilatory assist within 12 months prior to enrollment); previous cardiac transplantation, or complex congenital heart disease. Planned cardiac resynchronization therapy; Heart failure due to any of the following: known infiltrative cardiomyopathy (e.g. amyloid, sarcoid, lymphoma, endomyocardial fibrosis), active myocarditis, constrictive pericarditis, cardiac tamponade, known genetic hypertrophic cardiomyopathy or obstructive hypertrophic cardiomyopathy, arrhythmogenic right ventricular cardiomyopathy/dysplasia, or uncorrected primary valvular disease; life expectancy of less than 2 years due to any non-cardiovascular condition, based on investigator's clinical judgement; inability of the patient, in the opinion of the investigator, to understand and/or comply with study medications, procedures and/or follow-up OR any conditions that, in the opinion of the investigator, may render the patient unable to complete the study; active malignancy requiring treatment (with the exception of basal cell or squamous cell carcinomas of the skin); acute or chronic liver disease with severe impairment of liver function (e.g., ascites, esophageal varices, coagulopathy); women of child-bearing potential (i.e. those who are not chemically or surgically sterilized or post-menopausal) not willing to use a medically accepted method of contraception considered reliable in the judgment of the investigator OR who have a positive pregnancy test at randomization OR who are breast-feeding; involvement in the planning and/or conduct of the study (applies to both AstraZeneca personnel and/or personnel at the study site); previous randomization in the present study; participation in another clinical study with an investigational product or device during the last month prior to enrollment. |
| **Baseline Age** | I: 71.8 (9.6); C: 71.5 (9.5) |
| **Sample Size (N)** | O: 6263; I: 3131; C: 3132 |
| **Intervention** | Dapagliflozin at a dose of 10 mg once daily. |
| **Control** | Placebo. |
| **Primary and Secondary Outcome(s)** | Primary: a composite of worsening heart failure, which was defined as either an unplanned hospitalization for heart failure or an urgent visit for heart failure, or cardiovascular death.    Secondary: total number of worsening heart failure events and cardiovascular deaths, the change from baseline in the total symptom score on the Kansas City Cardiomyopathy Questionnaire (KCCQ; scores range from 0 to 100, with higher scores indicating fewer symptoms and physical limitations) at month 8, cardiovascular death and death from any cause. |
| **Findings** | Dapagliflozin reduced the combined risk of worsening heart failure or cardiovascular death among patients with heart failure and a mildly reduced or preserved ejection fraction. |
| **Funding** | AstraZeneca. |

| **Author, Year, Trial** | Anker, 2021, **EMPEROR-Preserved** |
| --- | --- |
| **Question/Study Objective** | Evaluated the effects of sodium–glucose cotransporter 2 inhibition with empagliflozin on major heart failure outcomes in patients with heart failure and a preserved ejection fraction. |
| **Study Design** | Randomized controlled trial. |
| **Sample Selection** | Inclusion: men or women; ≥18 years; New York Heart Association functional class II–IV chronic heart failure and a left ventricular ejection fraction of more than 40%. The protocol required patients to have an N-terminal pro–B-type natriuretic peptide (NT-proBNP) level of more than 300 pg per mL or, for patients with atrial fibrillation at baseline, an NT-proBNP level of more than 900 pg per mL.  Exclusion: had a disorder that could change their clinical course, independent of heart failure; or if they had any condition that might jeopardize patient safety or limit their participation in the trial |
| **Baseline Age** | Mean (SD): I: 71.8 (9.3); C: 71.9 (9.6) |
| **Sample Size (N)** | I: 2997; C: 2991 |
| **Intervention** | Empagliflozin, 10 mg per day, in addition to usual therapy. |
| **Control** | Placebo |
| **Primary and Secondary Outcome(s)** | Primary: combined risk of cardiovascular death or hospitalization for heart failure  Secondary: occurrence of all adjudicated hospitalizations for heart failure, including first and recurrent events. Rate of decline in the eGFR during double-blind treatment |
| **Findings** | Empagliflozin reduced the combined risk of cardiovascular death or hospitalization for heart failure in patients with heart failure and a preserved ejection fraction, regardless of the presence or absence of diabetes. |
| **Funding** | Boehringer Ingelheim and Eli Lilly. |

| **Author, Year, Trial** | Voors, 2022, **EMPULSE** |
| --- | --- |
| **Question/Study Objective** | Investigated the effects of empagliflozin on renal function and renal events during hospital admission and early after discharge. Explored the effects of empagliflozin on mortality, heart failure events and quality of life across the eGFR spectrum. |
| **Study Design** | Randomized controlled trial. |
| **Sample Selection** | Inclusion: men or women aged at least 18 years (at least 21 years in Japan, being the age of legal consent) who were hospitalized with a primary diagnosis of acute heart failure with dyspnea on exertion or at rest, and at least two of the following: congestion on chest radiograph, rales on chest auscultation, clinically relevant edema (for example, at least 1+ on a 0–3+ scale), or an elevated jugular venous pressure. Systolic blood pressure of at least 100mmHg; no inotropic support for at least 24h; no symptoms of hypotension; and in the 6h prior to randomization no increase in the i.v. diuretic dose and no i.v. vasodilators including nitrates; required to have an NT-proBNP concentration of at least 1600pgml−1 or a B-type natriuretic peptide (BNP) concentration of at least 400pgml−1. Patients in atrial fibrillation were required to have an NT-proBNP concentration of at least 2400pgml−1 or a BNP concentration of at least 600pgml−1. Patients had to be treated with a minimum dose of 40mg (20mg for Japanese patients) i.v. furosemide or equivalent.  Exclusion: cardiogenic shock; pulmonary embolism, cerebrovascular accident or acute myocardial infarction as the primary trigger for the current hospitalization or in the preceding 90 days before randomization; current or expected cardiac transplantation, left ventricular assist device, or inotropic support; an estimated glomerular filtration rate (eGFR) less than 20mlmin−1 per 1.73m2 or requiring dialysis; and prior ketoacidosis. |
| **Baseline Age** | Mean (SD): 68.4 (13.3); Median (IQR): O: 71 (61–78); I: 71 (62–78); C: 70 (59–78) |
| **Sample Size (N)** | O: 530; I: 265; C: 265 |
| **Intervention** | Empagliflozin 10 mg once daily. |
| **Control** | Placebo |
| **Primary and Secondary Outcome(s)** | Primary: clinical benefit, defined as a hierarchical composite of (i) time to death from any cause, (ii) number of heart failure events, (iii) time to first heart failure event, or (iv) a 5 point or greater difference in change from baseline in the Kansas City Cardiomyopathy Questionnaire total symptom score (KCCQ-TSS) at 90 days. Clinical benefit was assessed using a win ratio  Secondary: mortality, heart failure events and quality of life |
| **Findings** | In patients hospitalized for acute heart failure, empagliflozin caused an early modest decline in renal function which was no longer evident after 90 days. Acute renal events were similar in both groups. The clinical benefit of empagliflozin was consistent regardless of baseline renal function. |
| **Funding** | Boehringer Ingelheim and Eli Lilly and Company Diabetes Alliance. |

**Supplemental Table S2**: Risk of Bias for included studies for SGLT2i (N=14)

L=Low; U=Unclear; H=High

| **Study** | **SEQUENCE GENERATION** | **ALLOCATION CONCEALMENT** | **BLINDING OF PATIENTS AND PERSONNEL** | **BLINDING OUTCOME ASSESSMENT** | **INCOMPLETE OUTCOME DATA** | **SELECTIVE REPORTING** | **OTHER BIAS** |
| --- | --- | --- | --- | --- | --- | --- | --- |
| SOLOIST | L | U | U | U | H | H | L |
| DAPA-CKD | L | L | L | L | L | L | H |
| EMPEROR-REDUCED | U | U | L | L | L | L | L |
| DAPA-HF | L | U | U | U | L | L | U |
| DECALRE-TIMI | U | U | U | U | H | L | U |
| CREDENCE | L | L | L | L | L | L | U |
| CANVAS | L | U | L | U | L | L | U |
| EMPA-REG | L | U | U | L | L | L | L |
| VERTIS-CV | L | U | U | U | H | L | U |
| SCORED | U | U | U | U | L | L | U |
| EMPEROR-PRESERVED | L | L | U | U | L | L | U |
| EMPULSE | L | U | U | U | L | L | U |
| DELIVER | L | L | L | U | L | L | U |
| EMPA-KIDNEY | L | U | U | U | L | L | L |

**Supplemental Table S3: GRADE evidence rating**: ***Sodium glucose co-transporter 2 (SGLT-2) inhibitors compared to control for Cardio-renal Risk Reduction.***

| **Certainty assessment** | | | | | | | **№ of events / № of patients; Event rate** | | **Effect** | | **Certainty** | **Importance** |
| --- | --- | --- | --- | --- | --- | --- | --- | --- | --- | --- | --- | --- |
| **№ of studies** | **Study design** | **Risk of bias** | **Inconsistency** | **Indirectness** | **Imprecision** | **Other** | **SGLT-2 inhibitors** | **Control** | **Relative (95% CI)** | **Absolute (95% CI)** |  |  |
| **Cardiovascular Mortality - Overall (follow-up: range 9 months to 4.2 years)** | | | | | | | | | | | | |
| 13^a^ | randomised trials | serious^b^ | not serious^c^ | not serious | not serious^d^ | none^e^ | 2305/48485 (4.8%) | 2277/41928 (5.4%) | **HR 0.86** (0.81 to 0.92) | **7 fewer per 1,000** (from 10 fewer to 4 fewer) | ⨁⨁⨁◯ Moderate | CRITICAL |
| **Cardiovascular Mortality - Type 2 Diabetes (ASCVD / high CVD risk) (follow-up: range 9 months to 4.2 years)** | | | | | | | | | | | | |
| 12^f^ | randomised trials | serious^g^ | not serious^c^ | not serious | not serious^d^ | none^e^ | 1841/39166 (4.7%) | 1741/32598 (5.3%) | **HR 0.86** (0.80 to 0.93) | **7 fewer per 1,000** (from 10 fewer to 4 fewer) | 12^f^ | randomised trials |

| **Cardiovascular Mortality - Chronic Kidney disease (follow-up: range 1.3 years to 4.2 years)** | | | | | | | | | | | | |
| --- | --- | --- | --- | --- | --- | --- | --- | --- | --- | --- | --- | --- |
| 10^h^ | randomised trials | serious^i^ | not serious^c^ | not serious | not serious^d^ | none^e^ | 947/19623 (4.8%) | 1041/18803 (5.5%) | **HR 0.85** (0.78 to 0.93) | **8 fewer per 1,000** (from 12 fewer to 4 fewer) | ⨁⨁⨁◯ Moderate | CRITICAL |
| **Cardiovascular Mortality - Heart failure preserved EF (HFpEF) (follow-up: range 2.1 years to 4.2 years)** | | | | | | | | | | | | |
| 4^j^ | randomised trials | serious^k^ | not serious^c^ | not serious | serious^l^ | none^m^ | 551/7470 (7.4%) | 564/7104 (7.9%) | **HR 0.96** (0.82 to 1.14) | **3 fewer per 1,000** (from 14 fewer to 11 more) | ⨁⨁◯◯ Low | CRITICAL |
| **Cardiovascular Mortality - Heart failure reduced EF (HFrEF) (follow-up: range 1.3 years to 4.2 years)** | | | | | | | | | | | | |
| 4^n^ | randomised trials | serious^k^ | not serious^c^ | not serious | not serious^d^ | none^m^ | 481/4873 (9.9%) | 543/4750 (11.4%) | **HR 0.84** (0.71 to 0.98) | **17 fewer per 1,000** (from 32 fewer to 2 fewer) | ⨁⨁⨁◯ Moderate | CRITICAL |

| **Any cause Mortality - Overall (follow-up: range 9 months to 4.2 years)** | | | | | | | | | | | | |
| --- | --- | --- | --- | --- | --- | --- | --- | --- | --- | --- | --- | --- |
| 13^a^ | randomised trials | serious^b^ | not serious^c^ | not serious | not serious^d^ | none^e^ | 3808/48485 (7.9%) | 3718/41928 (8.9%) | **HR 0.88** (0.83 to 0.94) | **10 fewer per 1,000** (from 14 fewer to 5 fewer) | ⨁⨁⨁◯ Moderate | CRITICAL |
| **Any cause Mortality - Type 2 Diabetes (ASCVD / high CVD risk) (follow-up: range 9 months to 4.2 years)** | | | | | | | | | | | | |
| 11^o^ | randomised trials | serious^p^ | not serious^c^ | not serious | not serious^d^ | none^e^ | 2842/38239 (7.4%) | 2664/31669 (8.4%) | **HR 0.87** (0.81 to 0.94) | **11 fewer per 1,000** (from 15 fewer to 5 fewer) | ⨁⨁⨁◯ Moderate | CRITICAL |
| **Any cause Mortality - Chronic Kidney disease (follow-up: range 1.3 years to 4.2 years)** | | | | | | | | | | | | |
| 8^q^ | randomised trials | serious^r^ | not serious^c^ | not serious | not serious^d^ | none^m^ | 1204/17251 (7.0%) | 1452/16613 (8.7%) | **HR 0.82** (0.75 to 0.90) | **15 fewer per 1,000** (from 21 fewer to 8 fewer) | ⨁⨁⨁◯ Moderate | CRITICAL |

| **Any cause Mortality - Heart failure preserved EF (HFpEF) (follow-up: range 2.1 years to 4.2 years)** | | | | | | | | | | | | |
| --- | --- | --- | --- | --- | --- | --- | --- | --- | --- | --- | --- | --- |
| 4^j^ | randomised trials | serious^k^ | not serious^c^ | not serious | serious^l^ | none^m^ | 1066/7470 (14.3%) | 1064/7104 (15.0%) | **HR 0.97** (0.89 to 1.06) | **4 fewer per 1,000** (from 15 fewer to 8 more) | ⨁⨁◯◯ Low | CRITICAL |
| **Any cause Mortality - Heart failure reduced EF (HFrEF) (follow-up: range 1.3 years to 4.2 years)** | | | | | | | | | | | | |
| 4^n^ | randomised trials | serious^k^ | not serious^c^ | not serious | not serious^d^ | none^m^ | 617/4873 (12.7%) | 690/4750 (14.5%) | **HR 0.84** (0.72 to 0.97) | **22 fewer per 1,000** (from 38 fewer to 4 fewer) | ⨁⨁⨁◯ Moderate | CRITICAL |
| **Hospitalization due to heart failure - Overall (follow-up: range 9 months to 4.2 years)** | | | | | | | | | | | | |
| 12^s^ | randomised trials | serious^t^ | not serious^u^ | not serious | not serious^d^ | none^e^ | 2222/45181 (4.9%) | 2907/38623 (7.5%) | **HR 0.70** (0.66 to 0.73) | **22 fewer per 1,000** (from 25 fewer to 20 fewer) | ⨁⨁⨁◯ Moderate | CRITICAL |

| **Hospitalization due to heart failure - Type 2 Diabetes (ASCVD / high CVD risk) (follow-up: range 9 months to 4.2 years)** | | | | | | | | | | | | |
| --- | --- | --- | --- | --- | --- | --- | --- | --- | --- | --- | --- | --- |
| 11^v^ | randomised trials | serious^w^ | not serious^u^ | not serious | not serious^d^ | none^e^ | 1748/37711 (4.6%) | 2228/31147 (7.2%) | **HR 0.70** (0.65 to 0.75) | **21 fewer per 1,000** (from 24 fewer to 17 fewer) | ⨁⨁⨁◯ Moderate | CRITICAL |
| **Hospitalization due to heart failure - Chronic Kidney disease (follow-up: range 1.3 years to 4.2 years)** | | | | | | | | | | | | |
| 10^x^ | randomised trials | serious^p^ | not serious^u^ | not serious | not serious^d^ | none^e^ | 1158/18285 (6.3%) | 1612/16641 (9.7%) | **HR 0.65** (0.59 to 0.72) | **33 fewer per 1,000** (from 39 fewer to 26 fewer) | ⨁⨁⨁◯ Moderate | CRITICAL |
| **Hospitalization due to heart failure - Heart failure preserved EF (HFpEF) (follow-up: range 2.0 years to 4.2 years)** | | | | | | | | | | | | |
| 4^j^ | randomised trials | serious^k^ | not serious^u^ | not serious | not serious^d^ | none^m^ | 667/7470 (8.9%) | 856/7104 (12.0%) | **HR 0.74** (0.67 to 0.82) | **30 fewer per 1,000** (from 38 fewer to 21 fewer) | ⨁⨁⨁◯ Moderate | CRITICAL |

| **Hospitalization due to heart failure - Heart failure reduced EF (HFrEF) (follow-up: range 1.3 years to 4.2 years)** | | | | | | | | | | | | |
| --- | --- | --- | --- | --- | --- | --- | --- | --- | --- | --- | --- | --- |
| 4^n^ | randomised trials | serious^k^ | not serious^u^ | not serious | not serious^d^ | none^m^ | 551/4873 (11.3%) | 749/4750 (15.8%) | **HR 0.69** (0.64 to 0.75) | **46 fewer per 1,000** (from 54 fewer to 37 fewer) | ⨁⨁⨁◯ Moderate | CRITICAL |
| **Non-fatal myocardial infarction - Overall (follow-up: range 2.6 years to 4.2 years)** | | | | | | | | | | | | |
| 5^y^ | randomised trials | serious^z^ | not serious^u^ | not serious | not serious^c^ | none^m^ | 1211/26765 (4.5%) | 994/20204 (4.9%) | **HR 0.90** (0.83 to 0.98) | **5 fewer per 1,000** (from 8 fewer to 1 fewer) | ⨁⨁⨁◯ Moderate | CRITICAL |
| **Non-fatal myocardial infarction - Type 2 Diabetes (ASCVD / high CVD risk) (follow-up: range 2.6 years to 4.2 years)** | | | | | | | | | | | | |
| 5^y^ | randomised trials | serious^z^ | not serious^u^ | not serious | not serious^d^ | none^m^ | 1211/26765 (4.5%) | 994/20204 (4.9%) | **HR 0.90** (0.83 to 0.98) | **5 fewer per 1,000** (from 8 fewer to 1 fewer) | ⨁⨁⨁◯ Moderate | CRITICAL |

| **Non-fatal myocardial infarction - Chronic Kidney disease (follow-up: range 2.6 years to 4.2 years)** | | | | | | | | | | | | |
| --- | --- | --- | --- | --- | --- | --- | --- | --- | --- | --- | --- | --- |
| 3^aa^ | randomised trials | serious^ab^ | not serious^u^ | not serious | not serious^ac^ | none^m^ | 155/3918 (4.0%) | 197/3787 (5.2%) | **HR 0.77** (0.62 to 0.95) | **12 fewer per 1,000** (from 19 fewer to 3 fewer) | ⨁⨁⨁◯ Moderate | CRITICAL |
| **Non-fatal stroke - Overall (follow-up: range 2.6 years to 4.2 years)** | | | | | | | | | | | | |
| 5^y^ | randomised trials | serious^z^ | not serious^c^ | not serious | serious^l^ | none^m^ | 744/26765 (2.8%) | 560/20204 (2.8%) | **HR 0.99** (0.88 to 1.11) | **0 fewer per 1,000** (from 3 fewer to 3 more) | ⨁⨁◯◯ Low | CRITICAL |
| **Non-fatal stroke - Type 2 Diabetes (ASCVD / high CVD risk) (follow-up: range 2.6 years to 4.2 years)** | | | | | | | | | | | | |
| 5^y^ | randomised trials | serious^z^ | not serious^c^ | not serious | serious^l^ | none^m^ | 744/26765 (2.8%) | 560/20204 (2.8%) | **HR 0.99** (0.88 to 1.11) | **0 fewer per 1,000** (from 3 fewer to 3 more) | ⨁⨁◯◯ Low | CRITICAL |

| **Non-fatal stroke - Chronic Kidney disease (follow-up: range 2.6 years to 4.2 years)** | | | | | | | | | | | | |
| --- | --- | --- | --- | --- | --- | --- | --- | --- | --- | --- | --- | --- |
| 3^aa^ | randomised trials | serious^ab^ | serious^ad^ | not serious | serious^ae^ | none^m^ | 103/3918 (2.6%) | 129/3787 (3.4%) | **HR 0.78** (0.49 to 1.25) | **7 fewer per 1,000** (from 17 fewer to 8 more) | ⨁◯◯◯ Very low | CRITICAL |
| **CV mortality, non-fatal MI or stroke - Overall (follow-up: range 9 months to 4.2 years)** | | | | | | | | | | | | |
| 9^af^ | randomised trials | serious^ag^ | not serious^c^ | not serious | not serious^d^ | none^e^ | 3578/38121 (9.4%) | 3280/31567 (10.4%) | **HR 0.89** (0.84 to 0.93) | **11 fewer per 1,000** (from 16 fewer to 7 fewer) | ⨁⨁⨁◯ Moderate | CRITICAL |
| **CV mortality, non-fatal MI or stroke - Type 2 Diabetes (ASCVD / high CVD risk) (follow-up: range 9 months to 4.2 years)** | | | | | | | | | | | | |
| 7^ah^ | randomised trials | serious^ai^ | not serious^c^ | not serious | not serious^d^ | none^m^ | 3246/32665 (9.9%) | 2924/26110 (11.2%) | **HR 0.88** (0.82 to 0.93) | **13 fewer per 1,000** (from 19 fewer to 7 fewer) | ⨁⨁⨁◯ Moderate | CRITICAL |

| **CV mortality, non-fatal MI or stroke - Chronic Kidney disease (follow-up: range 1.4 years to 4.2 years)** | | | | | | | | | | | | |
| --- | --- | --- | --- | --- | --- | --- | --- | --- | --- | --- | --- | --- |
| 6^aj^ | randomised trials | serious^z^ | not serious^u^ | not serious | not serious^d^ | none^m^ | 1178/14439 (8.2%) | 1259/13497 (9.3%) | **HR 0.85** (0.78 to 0.92) | **13 fewer per 1,000** (from 20 fewer to 7 fewer) | ⨁⨁⨁◯ Moderate | CRITICAL |
| **CV mortality or HF hospitalization - Overall (follow-up: range 3 months to 4.2 years)** | | | | | | | | | | | | |
| 14^a^ | randomised trials | serious^b^ | not serious^c^ | not serious | not serious^d^ | none^e^ | 4218/48750 (8.7%) | 4818/42193 (11.4%) | **HR 0.77** (0.74 to 0.81) | **25 fewer per 1,000** (from 28 fewer to 21 fewer) | ⨁⨁⨁◯ Moderate | CRITICAL |
| **CV mortality or HF hospitalization - Type 2 Diabetes (ASCVD / high CVD risk) (follow-up: range 9 months to 4.2 years)** | | | | | | | | | | | | |
| 12^s^ | randomised trials | serious^t^ | not serious^c^ | not serious | not serious^d^ | none^e^ | 3314/39166 (8.5%) | 3694/32598 (11.3%) | **HR 0.77** (0.73 to 0.80) | **25 fewer per 1,000** (from 29 fewer to 22 fewer) | ⨁⨁⨁◯ Moderate | CRITICAL |

| **CV mortality or HF hospitalization - Chronic Kidney disease (follow-up: range 3 months to 4.2 years)** | | | | | | | | | | | | |
| --- | --- | --- | --- | --- | --- | --- | --- | --- | --- | --- | --- | --- |
| 12^ak^ | randomised trials | serious^g^ | not serious^u^ | not serious | not serious^d^ | none^e^ | 2015/20468 (9.8%) | 2618/20403 (12.8%) | **HR 0.75** (0.70 to 0.79) | **30 fewer per 1,000** (from 37 fewer to 26 fewer) | ⨁⨁⨁◯ Moderate | CRITICAL |
| **CV mortality or HF hospitalization - Heart failure preserved EF (HFpEF) (follow-up: range 9 months to 4.2 years)** | | | | | | | | | | | | |
| 6^al^ | randomised trials | serious^k^ | not serious^c^ | not serious | not serious^d^ | none^m^ | 1237/8849 (14.0%) | 1476/8467 (17.4%) | **HR 0.79** (0.73 to 0.86) | **34 fewer per 1,000** (from 44 fewer to 22 fewer) | ⨁⨁⨁◯ Moderate | CRITICAL |
| **CV mortality or HF hospitalization - Heart failure reduced EF (HFrEF) (follow-up: range 9 months to 4.2 years)** | | | | | | | | | | | | |
| 6^am^ | randomised trials | serious^k^ | not serious^u^ | not serious | not serious^d^ | none^m^ | 1031/5739 (18.0%) | 1294/5642 (22.9%) | **HR 0.75** (0.69 to 0.81) | **52 fewer per 1,000** (from 65 fewer to 39 fewer) | ⨁⨁⨁◯ Moderate | CRITICAL |
| **Kidney composite outcome - Overall (follow-up: range 1.3 years to 4.2 years)** | | | | | | | | | | | | |
| 12^an^ | randomised trials | serious^t^ | not serious^c^ | not serious | not serious^d^ | none^e^ | 1899/47835 (4.0%) | 2177/41303 (5.3%) | **HR 0.68** (0.60 to 0.77) | **17 fewer per 1,000** (from 21 fewer to 12 fewer) | ⨁⨁⨁◯ Moderate | CRITICAL |
| **Kidney composite outcome - Type 2 Diabetes (ASCVD / high CVD risk) (follow-up: range 1.3 years to 4.2 years)** | | | | | | | | | | | | |
| 12^an^ | randomised trials | serious^t^ | not serious^c^ | not serious | not serious^d^ | none^e^ | 1568/40041 (3.9%) | 1755/33489 (5.2%) | **HR 0.67** (0.59 to 0.75) | **17 fewer per 1,000** (from 21 fewer to 13 fewer) | ⨁⨁⨁◯ Moderate | CRITICAL |
| **Kidney composite outcome - Chronic Kidney disease (follow-up: range 1.3 years to 4.2 years)** | | | | | | | | | | | | |
| 12^an^ | randomised trials | serious^t^ | not serious^c^ | not serious | not serious^d^ | none^e^ | 1134/23746 (4.8%) | 1479/21924 (6.7%) | **HR 0.68** (0.60 to 0.76) | **21 fewer per 1,000** (from 26 fewer to 16 fewer) | ⨁⨁⨁◯ Moderate | CRITICAL |

| **Kidney composite outcome - Heart failure preserved EF (HFpEF) (follow-up: mean 2.1 years)** | | | | | | | | | | | | |
| --- | --- | --- | --- | --- | --- | --- | --- | --- | --- | --- | --- | --- |
| 2^ao^ | randomised trials | serious^k^ | not serious | not serious | serious^ae^ | none^m^ | 187/6128 (3.1%) | 185/6122 (3.0%) | **HR 1.00** (0.82 to 1.23) | **0 fewer per 1,000** (from 5 fewer to 7 more) | ⨁⨁◯◯ Low | CRITICAL |
| **Kidney composite outcome - Heart failure reduced EF (HFrEF) (follow-up: range 1.3 years to 1.5 years)** | | | | | | | | | | | | |
| 2^ap^ | randomised trials | serious^aq^ | not serious^c^ | not serious | not serious^ac^ | none^m^ | 58/4236 (1.4%) | 97/4238 (2.3%) | **HR 0.59** (0.42 to 0.83) | **9 fewer per 1,000** (from 13 fewer to 4 fewer) | ⨁⨁⨁◯ Moderate | CRITICAL |
| **Serious AEs leading to study discontinuation (follow-up: range 3 months to 4.2 years)** | | | | | | | | | | | | |
| 13^ar^ | randomised trials | serious^b^ | serious^ad^ | not serious | serious^l^ | none^e^ | 3785/46859 (8.1%) | 2875/40302 (7.1%) | **RR 1.04** (0.96 to 1.12) | **2 more per 1,000** (from 3 fewer to 9 more) | ⨁◯◯◯ Very low | CRITICAL |

**CI:** confidence interval; **HR:** hazard Ratio; **RR:** risk ratio

#### Explanations

a. CANVAS; CREDENCE; DAPA-CKD; DAPA-HF; DECLARE–TIMI; DELIVER; EMPA-REG; EMPA-KIDNEY; EMPEROR-Preserved; EMPEROR-Reduced; SCORED; SOLOIST-WHF; VERTIS CV; EMPULSE (time to event data for CV mortality or HF hospitalization only) *

b. 11 out of 13 studies were rated as unclear risk of bias with concerns and lack of reporting on randomization, and allocation concealment procedures; blinding of participants and outcome assessment; incomplete/selective outcome reporting; and other sources of bias such as industry funding.

c. The direction of effect is consistent and confidence intervals overlap with minimal to moderate statistical heterogeneity observed across studies.

d. The sample size and number of events are adequate (=>300) in each arm. The effect estimate is precise with narrow confidence intervals.

e. No noticeable asymmetry in the funnel plot and the test for funnel plot asymmetry was non-significant (p > 0.05).

f. CANVAS; CREDENCE; DAPA-CKD; DAPA-HF; DECLARE–TIMI; DELIVER; EMPA-REG; EMPEROR-Preserved; EMPEROR-Reduced; SCORED; SOLOIST-WHF; VERTIS CV.

g. 10 out of 12 studies were rated as unclear risk of bias with concerns and lack of reporting on randomization, and allocation concealment procedures; blinding of participants and outcome assessment; incomplete/selective outcome reporting; and other sources of bias such as industry funding.

h. CANVAS; CREDENCE; DAPA-CKD; DAPA-HF; DECLARE-TIMI; DELIVER; EMPA-REG; EMPA-KIDNEY; EMPEROR-Reduced; SCORED.

i. 8 out of 10 studies were rated as unclear risk of bias with concerns and lack of reporting on randomization, and allocation concealment procedures; blinding of participants and outcome assessment; incomplete/selective outcome reporting; and other sources of bias such as industry funding.

j. DECLARE-TIMI; DELIVER; EMPEROR-Preserved; VERTIS CV.

k. Studies were rated as unclear risk of bias with concerns and lack of reporting on randomization, and allocation concealment procedures; blinding of participants and outcome assessment; incomplete/selective outcome reporting; and other sources of bias such industry funding.

l. The sample size and number of events are adequate (=>300) in each arm, however, the effect estimate is imprecise with wide confidence intervals that do not rule out a null effect or harm.

m. Too few studies (n<10) to assess publication bias.

n. DAPA-HF; DECLARE-TIMI; EMPEROR-Reduced; VERTIS CV.

o. CANVAS; CREDENCE; DAPA-CKD; DAPA-HF; DECLARE–TIMI; DELIVER; EMPA-REG; EMPEROR-Preserved; SCORED; SOLOIST-WHF; VERTIS CV.

p. 9 out of 11 studies were rated as unclear risk of bias with concerns and lack of reporting on randomization, and allocation concealment procedures; blinding of participants and outcome assessment; incomplete/selective outcome reporting; and other sources of bias such as industry funding.

q. CREDENCE; DAPA-CKD; DAPA-HF; DECLARE-TIMI; DELIVER; EMPA-REG; EMPEROR-Reduced; SCORED.

r. 6 out of 8 studies were rated as unclear risk of bias with concerns and lack of reporting on randomization, and allocation concealment procedures; blinding of participants and outcome assessment; incomplete/selective outcome reporting; and other sources of bias such as industry funding.

s. CANVAS; CREDENCE; DAPA-CKD; DAPA-HF; DECLARE–TIMI; DELIVER; EMPA-REG; EMPEROR-Preserved; EMPEROR-Reduced; SCORED; SOLOIST-WHF; VERTIS CV.

t. 10 out of 12 studies were rated as unclear risk of bias with concerns and lack of reporting on randomization, and allocation concealment procedures; blinding of participants and outcome assessment; incomplete/selective outcome reporting; and other sources of bias such as industry funding.

u. The direction of effect is consistent and confidence intervals overlap with minimal statistical heterogeneity observed across studies.

v. CANVAS; CREDENCE; DAPA-HF; DECLARE–TIMI; DELIVER; EMPA-REG; EMPEROR-Preserved; EMPEROR-Reduced; SCORED; SOLOIST-WHF; VERTIS CV.

w. 10 out of 11 studies were rated as unclear risk of bias with concerns and lack of reporting on randomization, and allocation concealment procedures; blinding of participants and outcome assessment; incomplete/selective outcome reporting; and other sources of bias such as industry funding.

x. CANVAS; CREDENCE; DAPA-CKD; DAPA-HF; DECLARE-TIMI; DELIVER; EMPA-REG; EMPEROR-Reduced; SCORED; VERTIS CV.

y. CANVAS; CREDENCE; DECLARE–TIMI; EMPA-REG; VERTIS CV.

z. 5 out of 6 studies were rated as unclear risk of bias with concerns and lack of reporting on randomization, and allocation concealment procedures; blinding of participants and outcome assessment; incomplete/selective outcome reporting; and other sources of bias such as industry funding.

aa. CANVAS; CREDENCE; DECLARE-TIMI.

ab. 2 out of 3 studies were rated as unclear risk of bias with concerns and lack of reporting on randomization, and allocation concealment procedures; blinding of participants and outcome assessment; incomplete/selective outcome reporting; and other sources of bias such as industry funding.

ac. The sample size is adequate (=>300) in each arm and the number of events is < 300 in each arm. However, the effect estimate is precise with narrow confidence intervals.

ad. The direction of effect is not consistent with high heterogeneity observed across studies.

ae. The sample size is adequate (=>300) in each arm, but the number of events is < 300 in each arm, and the effect estimate is imprecise with wide confidence intervals that do not rule out a null effect or harm.

af. CANVAS; CREDENCE; DAPA-CKD; DECLARE–TIMI; DELIVER; EMPA-REG; SCORED; SOLOIST-WHF; VERTIS CV.

ag. 8 out of 9 studies were rated as unclear risk of bias with concerns and lack of reporting on randomization, and allocation concealment procedures; blinding of participants and outcome assessment; incomplete/selective outcome reporting; and other sources of bias such as industry funding.

ah. CANVAS; CREDENCE; DECLARE–TIMI; EMPA-REG; SCORED; SOLOIST-WHF; VERTIS CV.

ai. 6 out of 7 studies were rated as unclear risk of bias with concerns and lack of reporting on randomization, and allocation concealment procedures; blinding of participants and outcome assessment; incomplete/selective outcome reporting; and other sources of bias such industry funding.

aj. CANVAS; DAPA-CKD; DECLARE-TIMI; DELIVER; EMPA-REG; EMPA-REG; SCORED.

ak. CANVAS; CREDENCE; DAPA-CKD; DAPA-HF; DECLARE-TIMI; DELIVER; EMPA-KIDNEY; EMPEROR-Preserved; EMPEROR-Reduced; EMPULSE; SCORED; SOLOIST-WHF.

al. DECLARE-TIMI; DELIVER; EMPEROR-Preserved; SCORED; SOLOIST-WHF; VERTIS CV.

am. DAPA-HF; DECLARE-TIMI; EMPEROR-Reduced; SCORED; SOLOIST-WHF; VERTIS CV.

an. CANVAS; CREDENCE; DAPA-CKD; DAPA-HF; DECLARE–TIMI; DELIVER; EMPA-REG; EMPA-KIDNEY; EMPEROR-Preserved; EMPEROR-Reduced; SCORED; VERTIS CV.

ao. DELIVER; EMPEROR-Preserved.

ap. DAPA-HF; EMPEROR-Reduced.

aq. Both studies were rated as unclear risk of bias with concerns and lack of reporting on randomization, and allocation concealment procedures; blinding of participants and outcome assessment; and other sources of bias such as industry funding.

ar. CANVAS; CREDENCE; DAPA-CKD; DAPA-HF; DECLARE–TIMI; DELIVER; EMPA-REG; EMPA-KIDNEY; EMPEROR-Preserved; EMPULSE; SCORED; SOLOIST-WHF; VERTIS CV.
